# Characterization of Repetitive DNA in *Saccharum officinarum* and *Saccharum spontaneum* by Genome Sequencing and Cytological Assays

**DOI:** 10.3389/fpls.2022.814620

**Published:** 2022-02-22

**Authors:** Kai Wang, Dong Xiang, Kai Xia, Bo Sun, Haris Khurshid, Ayman M. H. Esh, Hui Zhang

**Affiliations:** ^1^School of Life Sciences, Nantong University, Nantong, China; ^2^Guangxi Key Laboratory of Sugarcane Biology & Key Laboratory of Genetics, Breeding and Multiple Utilization of Crops, Ministry of Education, Fujian Agriculture and Forestry University, Fuzhou, China; ^3^Oilseeds Research Program, National Agricultural Research Centre, Islamabad, Pakistan; ^4^Sugar Crops Research Institute, Agriculture Research Center, Giza, Egypt

**Keywords:** *Saccharum*, repetitive DNA, FISH, polyploid, centromeric retrotransposon

## Abstract

In most plant species, DNA repeated elements such as satellites and retrotransposons are composing the majority of their genomes. *Saccharum officinarum* (2*n* = 8*x* = 80) and *S. spontaneum* (2*n* = 40–128) are the two fundamental donors of modern sugarcane cultivars. These two species are polyploids with large genome sizes and are enriched in repetitive elements. In this work, we adopted a *de novo* strategy to isolate highly repetitive and abundant sequences in *S. officinarum* LA Purple and *S. spontaneum* SES208. The findings obtained from alignment to the genome assemblies revealed that the vast majority of the repeats (97.9% in LA Purple and 96.5% in SES208) were dispersed in the respective genomes. Fluorescence *in situ* hybridization assays were performed on 27 representative repeats to investigate their distributions and abundances. The results showed that the copies of some highly repeated sequences, including rDNA and centromeric or telomeric repeats, were underestimated in current genome assemblies. The analysis of the raw read mapping strategy showed more copy numbers for all studied repeats, suggesting that copy number underestimation is common for highly repeated sequences in current genome assemblies of LA Purple and SES208. In addition, the data showed that the centromeric retrotransposons in all SES208 centromeres were absent in certain *S. spontaneum* clones with different ploidies. This rapid turnover of centromeric DNA in sugarcane provides new clues regarding the pattern of centromeric retrotransposon formation and accumulation.

## Introduction

In eukaryotes, repetitive sequences (also referred to as repeats) in genomes are ubiquitous (Treangen and Salzberg, 2012; Wicker et al., 2018) and often present as a major component (Feschotte and Pritham, 2007). Repetitive elements, for example, may account for more than two-thirds of the human genome (de Koning et al., 2011) and up to 80% in wheat (Garbus et al., 2015). Despite their pervasiveness, it is still a lot to be understood about the mechanisms by which repetitive elements emerge and rapidly accumulate.

Tandem repeating DNAs and transposable elements (TEs) are the two typical repeats in eukaryotes. In plants, tandem repeats can be arranged in tandem arrays of thousands of neighboring monomers, reaching megabase size in the genome (Flavell, 1986; Charlesworth et al., 1994; Kejnovsky et al., 2009; Gong et al., 2012; Mehrotra and Goyal, 2014; Biscotti et al., 2015; Garrido-Ramos, 2015). There are three well-known types of tandem repeats: centromeric satellite repeats, telomeric satellite repeats, and ribosomal DNA (5S and 45S rDNAs) (de Koning et al., 2011; Biscotti et al., 2015). TEs constitute the most abundant component of many genomes, ranging from 10 to 85% (Rebollo et al., 2012). Long terminal repeat (LTR)-type retrotransposons are typically the most type of TE in plants, especially for species with large genome sizes, such as sugarcane, wheat, maize, and cotton (Schnable et al., 2009; Appels et al., 2018; Zhang et al., 2018; Yang et al., 2020). LTR-type retrotransposons can move *via* ‘copy and paste’ mechanisms and was enriched in the centromeres of plants (Presting, 2018).

Repetitive sequences have a large influence on genome structure, function and evolution. In cotton, *Gossypium arboreum* and *G. raimondii* were derived from a common ancestor approximately 5–10 million years ago (Wendel, 1989; Li et al., 2014) and rapid proliferation of TEs enlarged the genome of *G. arboretum*, leading to twice as large as the genome size of *G. raimondii* (Paterson et al., 2012; Li et al., 2014). Moreover, the shift and rapid proliferation of TEs between subgenomes in polyploid cotton had a deep impact on centromere DNA composition (Han et al., 2016). In addition, a large portion of maize open chromatins, which frequently contain *cis*-regulatory DNA elements, were derived from TEs (Zhao et al., 2018; Marand et al., 2021). Moreover, Lu et al. (2019) reported that the majority of distal open chromatins have been moved away from their target genes by TE proliferation in plants. Therefore, these reports indicate that TEs play a major role in either transcriptional regulation or the formation of distal regulatory elements in plants. TEs also are an extensive source of mutations and genetic polymorphisms. In *D. melanogaster*, more than half of all known phenotypic mutants isolated in the laboratory are caused by spontaneous insertions of a wide variety of TEs (Eickbush and Furano, 2002). Transposition events are also common in plants and have been applied widely to generate mutagenic lines (Kumar and Bennetzen, 1999; Cui et al., 2013).

However, the ubiquity of repetitive sequences also complicates genomic analysis. The chromosomal regions enriched with tandem repeat often represent the final barriers to completing whole-genome sequencing. Although many eukaryotic genomes have been sequenced, most tandem repeat regions have yet to be finished (Bourque et al., 2018). Moreover, tandemly arranged repeats will impact repeat-harboring fragment assembly, which may consequently lead to underestimation of the copy or incorrect alignment in the genome assembly. Thus, isolation and characterization repeats with respect to their genome-wide distributions and abundances is very important for genomic studies.

Fluorescence *in situ* hybridization (FISH) is a powerful and unique tool for the physical mapping of DNA sequences. It displays visible information regarding the physical map position of sequence and is often the sole way to determine the abundance and distribution of repetitive sequence (Schwarzacher, 2003). In sugarcane, the whole-genome sequences of *S. spontaneum* clone SES208 (2*n* = 8*x* = 64) (Zhang et al., 2018) and modern cultivar R570 (Garsmeur et al., 2018) have been achieved. Recently, the genome of a *S. officinarum* clone LA Purple (2*n* = 8*x* = 80) was also assembled (Ming et al., unpublished). However, the contents and chromosomal distributions of repetitive sequences are still largely uncharacterized in these genomes. Here, a genome-wide scan was performed to detect repetitive sequences in the two autopolyploid sugarcane species *S. officinarum* (2*n* = 8*x* = 80) and *S. spontaneum.* By combining computational alignment and FISH assays, we obtained the composition and distribution of repeats in the two complex genomes.

## Materials and Methods

### Plant Materials

*Saccharum spontaneum* clones SES208 (2*n* = 8*x* = 64), Yunnan82-16 (2*n* = 8*x* = 64), Yunnan82-29 (2*n* = 10*x* = 80), Sichuan79-I-1 (2*n* = 11*x* = 88), Sichuan79-II-11 (2*n* = 12*x* = 96), Guizhou78-II-28 (2*n* = 13*x* = 104), and *S. officinarum* clone LA Purple (2*n* = 8*x* = 80) were used in this study. All of the plants were grown in the greenhouse with a 16 h light/8 h dark photoperiod at 30°C.

### Genome Sequencing and Repetitive Sequences Identification

For genome survey sequencing, DNAs from leaf tissues were extracted using Super Plant Genomic DNA Kit (TIANGEN cat. no. DP360, Beijing, China) according to the protocol provided by manufactory. The DNAs from leaf tissues were then used to construct sequencing library using the kit of NEBNext-Ultra™ DNA Library Prep Kit for Illumina (cat. no. E7370, San Diego, CA, United States). Genome sequencing was conducted using the Illumina HiSeq X platform. A total of ∼33 million 150-bp paired-end sequence reads were obtained from LA Purple and SES208, which accounts for ∼1.5x coverage of the respective genome size (6.7 Gb for LA Purple, 6.6 Gb for SES208). The sequence reads were first treated using Trimmomatic v.0.39 (Bolger et al., 2014) to remove low quality reads. The Q30 (1/1000 chance of an incorrect base) percentage of clean data was ∼97%, which was of sufficiently high for subsequent analyses. Sequence data for the LA Purple and SES208 can be found in the National Genomics Data Center data library under accession number PRJCA007170.

The repetitive DNA sequences from each clone were identified using the similarity-based clustering method (Novak et al., 2013). For a given genome, a total of 5 million randomly selected sequence reads (150 bp) were analyzed using web-based Galaxy RepeatExplorer software^1^ with the default parameters. The repeats were then identified and classified as individual repeat clusters based on their sequence similarity. The genome proportion of each repeat cluster was estimated based on the number of reads in each repeat cluster.

### Genomic Distribution and Copy Number Estimation

The genome assemblies of SES208 (Zhang et al., 2018) and LA Purple (Ming et al., unpublished) were applied in this study. To identify copies in the genome assembly, BLASTn analysis was performed to map the contig sequence to the reference genome. The significant BLASTn hits that had a sequence identity > 70% and coverage > 70% were retained and shown in the Integrative Genomics Viewer for distribution analysis. The copy number for each contig was calculated as total aligned length/contig length.

To estimate copy numbers using raw reads, 45 and 40 million reads that accounted for 1 × genome coverage of LA Purple and SES208, respectively, were randomly selected. These reads were then mapped to each contig sequence by BLASTn. The significant BLASTn hits (sequence identity > 70% and coverage > 70%) were retained, and the copy number for each contig was calculated as described above.

### Probe and Chromosome Spread Preparation

The probe DNAs were amplified by PCR from corresponding genomic DNAs of SES208 and LA Purple. Primers for each repeat were designed to the representative contig sequence (Supplementary Table 1). PCR products with an expected size were extracted from the gel and labeled with either digoxigenin-11-dUTP (Roche Diagnostics, Mannhei, Germany) or biotin-16-dUTP (Roche Diagnostics, Mannhei, Germany) using standard nick translation reactions.

Mitotic chromosome spreads of each sample were prepared as previously described (Meng et al., 2018). Briefly, root tips were harvested from sugarcane and treated in nitrous oxide at a pressure of 10.9 atm (∼160 psi) for 1–2 h, fixed in Carnoy’s fixative (3 ethanol:1 acetic acid) and stored at –20°C until use. Subsequently, the root tips were digested in an enzymatic solution with 2% cellulase (Yakult Pharmaceutical, Tokyo, Japan) and 1% pectolyase (Sigma Chemical, St. Louis, MO, United States) at 37°C for 1 h and then squashed with a cover slip. After the slides were frozen in liquid nitrogen, the cover slips were removed, and the slides were dehydrated with an ethanol series (70, 90, and 100%, 5 min each) prior to FISH assay.

### Fluorescence *in situ* Hybridization Assay

Fluorescence *in situ* hybridization was performed following published protocols (Meng et al., 2018). First, the chromosome slides were denatured in 70% formamide in 2x SSC at 70°C for 1 min and dehydrated in an ethanol series (70, 90, and 100%; 5 min each). The hybridization mixture (50% formamide, 10% dextran sulfate, 20× SSC, 50 ng labeled probe) was denatured at 90°C for 5 min. Next, the hybridization mixture was applied to the denatured chromosome slides and incubated for 12 h at 37°C. Then, the slides were washed in 2x SSC, 50% formamide in 2x SSC, and in 2x SSC at 42°C for 5 min each. Subsequently, digoxigenin- and biotin-labeled probes were detected using rhodamine-conjugated anti-digoxigenin (Roche Diagnostics, United States) and fluorescein-conjugated avidin (Life Technologies, United States), respectively. Chromosome slides were counterstained with 4′, 6′-diamidino-phenylindole (DAPI) in a VECTASHIELD antifade solution (Vector Laboratories, Burlingame, CA, United States). FISH signals were detected under an Olympus BX63 fluorescence microscope. Images were captured and merged by cellSens Dimension 1.9 software with an Olympus DP80 CCD camera. For image assay, 7–10 cells were analyzed. The final images were processed and adjusted by Adobe Photoshop CC software.

## Results

### Genome-Wide Identification of Repetitive DNA Sequences From *Saccharum officinarum* and *Saccharum spontaneum*

A total of 161,028 and 90,804 repeat clusters were obtained from LA Purple and SES208, respectively (Supplementary Figure 1), using RepeatExplorer. Among them, 338 and 305 clusters that accounted for 44.9 and 42.4% of the total 33 million genomic reads were relatively enriched in LA Purple and SES208, respectively (genome proportion > 0.01%) and were annotated to characterize the most repeat families.

**FIGURE 1:**
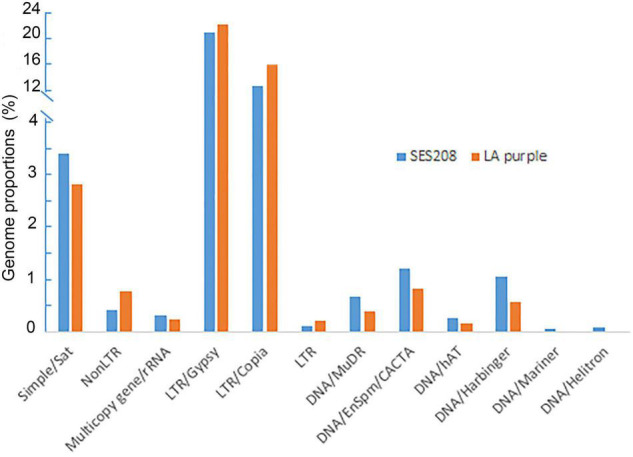
The composition and annotation of repetitive DNA in *S. officinarum* LA Purple and *S*. *spontaneum* clone SES208. Annotation and genome proportions of the 338 and 305 repeat clusters in *S. officinarum* LA Purple and *S*. *spontaneum* clone SES208, respectively.

These two species demonstrated highly similar of repetitive DNA compositions (Figure 1). LTR retrotransposons were the most abundant repeat families, accounting for 38.7 and 34.7% of the LA Purple and SES208 genomes, respectively (Figure 1). Among them, the Ty3/*gypsy* retrotransposons were the most enriched, representing 22.7% in LA Purple and 22.1% in SES208, followed by LTR/*copia*, accounting for 16.0% in LA Purple and 12.6% in SES208. We also observed satellite repeats from both genomes, representing 2.8 and 3.4% of LA Purple and SES208, respectively. Several types of DNA transposons were found in both genomes but accounted for relatively minor proportions of the genomes (<2%) (Figure 1).

### Genomic Distributions of Repetitive Sequences in *Saccharum officinarum* LA Purple

To analyze genomic distribution, a representative contig with the highest read depth in each of the 644 repeat clusters were selected for further analysis. The representative contigs were then computationally aligned to LA Purple and SES208 genome assemblies, respectively (Zhang et al., 2018; Ming et al., unpublished). For LA Purple, 96–18,173 copies were identified for each of the 339 repeats. Except for repeat LA1C934 (18,173 copies), all the repeats showed fewer than 6500 copies, suggesting less than one copy per megabase in the 6.80 Gb LA Purple genome. To facilitate analysis, 190 repeats with at least 320 copies (an average of four copies of each chromosome) in LA Purple were selected for further analysis (Supplementary Table 2).

**FIGURE 2:**
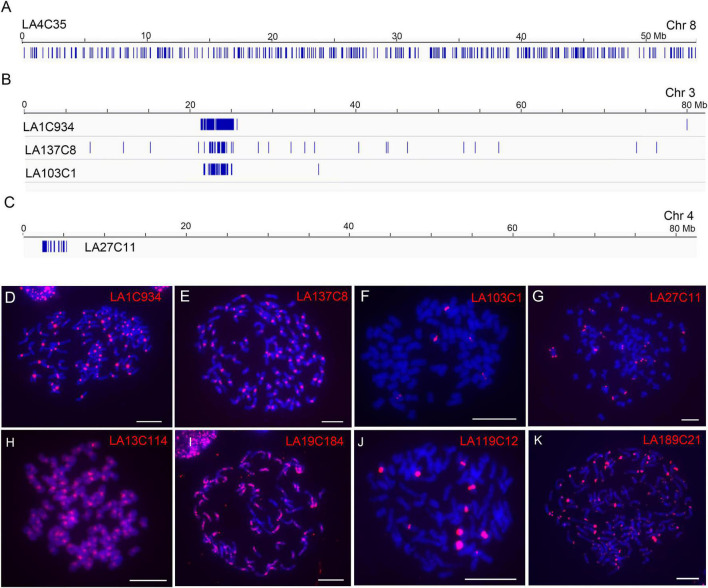
Characterization of genomic distributions of representative repeats in LA Purple. **(A–C)** Diagrams illustrate the distributions of repeats LA4C35, LA1C934, LA137C8, LA103C1, and LA27C1 in assembled pseudochromosomes. Blue bars represent homologous copies of the repeat. **(D–K)** FISH analyses of representative repeats in LA Purple. Eight repeats, LA1C934 **(D)**, LA137C8 **(E)**, LA103C1 **(F)**, LA27C11 **(G)**, LA13C114 **(H)**, LA19C184 **(I)**, LA119C12 **(J)**, and LA189C21 **(K)**, were mapped to the metaphase chromosomes. Bars = 10 μm.

By checking the distributions of each repeat, we found that 186 of the 190 repeats (97.9%) showed largely dispersed distributions (no apparent clustered copies) in the LA Purple genome assembly (Figure 2A, exemplified with repeat LA4C35), indicating the dominance of dispersed repeats in the LA Purple genome. For four repeats (LA1C934, LA137C8, LA103C1, and LA27C11), we observed clustered copies at some regions in the pseudochromosomes (Figures 2B,C). Intriguingly, the copy-enriched regions for repeats LA1C934, LA137C8 and LA103C1 were largely colocalized (Figure 2B), indicating that they had similar chromosome localization. For repeat LA27C11, copies were mainly concentrated in the distal ends in some pseudochromosomes (Figure 2C).

To further confirm the genomic distributions, these four non-dispersed repeats (LA1C934, LA137C8, LA103C1, and LA27C11) were PCR amplified and labeled for FISH. Centromeric signals from LA1C934, LA137C8, and LA103C1 were observed in FISH (Figures 2D–F). However, centromere signals from all chromosomes were detected from probes of LA1C934 and LA137C8 (Figures 2D,E), but only five centromeric signals were found with FISH for repeat LA103C1 (Figure 2F). Moreover, these five centromeric signals showed different signal intensities (two were large signals and the other three were very small) (Figure 2F), indicating highly different copies for these loci. Sequence annotation revealed that LA1C934 was a tandem repeated sequence with high sequence similarity (84%) to a centromere sequence of the *S. officinarum* clone (NCBI Sequence ID: MG708495). Both LA137C8 and LA103C1 were annotated as LTR retrotransposons (Table 1) but with no sequence similarity to each other. For repeat LA27C11, FISH results showed signals in the distal ends of ∼20 chromosomes (some signals were too weak to be detected consistently in all studied cells). However, sequence analysis showed no plant telomeric repeat array (TTTAGGG) (Watson and Riha, 2010) included in LA27C11, and no sequence with high similarity was found by BLASTn.

**TABLE 1:** Summary of repetitive sequences in LA Purple and SES208*^a^*.

Repeat	Cluster	Repeat classification	Distribution in genome assembly	FISH mapping	Copy numbers estimation		
					Genome assembly	Read mapping	Fold change
**Repeats of LA Purple**							
LA1C934	CL1Contig934	Simple/Sat	Non-dispersed	Centromeric signals on all chromosomes	18174	44408	2.4
LA137C8	CL137Contig8	LTR	Non-dispersed	Centromeric signals on all chromosomes	771	6729	8.7
LA103C1	CL103Contig1	LTR/*Gypsy*	Non-dispersed	Centromeric signals on five chromosomes	1168	3386	2.9
LA27C11	CL27Contig11	Simple/Sat	Non-dispersed	Distal ends of ∼20 chromosomes	2927	64493	22.0
LA38C21	CL38Contig21	LTR/*Gypsy*	Dispersed	Dispersed signals at all chromosomes	2455	10492	4.3
LA65C19	CL65Contig19	Harbinger	Dispersed	Dispersed signals at all chromosomes	1867	8253	4.4
LA4C35	CL4Contig35	LTR/*Copia*	Dispersed	Dispersed signals at all chromosomes	5612	40823	7.3
LA13C114	CL13Contig114	LTR/*Gypsy*	Dispersed	Dispersed signals at all chromosomes, strong signal at centromeric regions	3561	30877	8.7
LA19C184	CL19Contig184	LTR/*Gypsy*	Dispersed	Dispersed signals at part of chromosomes	3196	15628	4.9
LA119C12	CL119Contig12	LTR/*Gypsy*	Dispersed	Centromeric signals at ten chromosomes	983	6636	6.8
LA189C21	CL189Contig21	EnSpm/CACTA	Dispersed	Telomeric signals at all chromosomes	340	3144	9.2
LA143C8	CL143Contig8	MuDR	Dispersed	No detectable signal	681	1595	2.3

**Repeats of SES208**							

Se1C692	CL1Contig692	Simple/Sat	Non-dispersed	Centromeric signals on all chromosomes	35043	71582	2.0
Se50C34	CL50Contig34	Unknown	Non-dispersed	Centromeric signals on all chromosomes	2744	6276	2.3
Se144C9	CL144Contig9	LTR/*Gypsy*	Non-dispersed	Centromeric signals on all chromosomes	998	2719	2.7
Se147C14	CL147Contig14	LTR/*Gypsy*	Non-dispersed	Centromeric signals on all chromosomes	974	1797	1.8
Se207C4	CL207Contig4	LTR	Non-dispersed	Centromeric signals on all chromosomes	340	4281	12.6
Se12C28	CL12Contig28	LTR/*Gypsy*	Non-dispersed	Centromeric signals on ∼40 chromosomes	5561	12320	2.2
Se23C16	CL23Contig16	LTR/*Gypsy*	Non-dispersed	Centromeric signals on ∼40 chromosomes	4072	12304	3.0
Se157C13	CL157Contig13	Simple/Sat	Non-dispersed	Signals on the distal ends of 4 chromosomes	790	3266	4.1
Se27C99	CL27Contig99	LTR/*Copia*	Dispersed	Dispersed signals at all chromosomes	3797	21448	5.6
Se96C18	CL96Contig18	LTR/*Gypsy*	Dispersed	Dispersed signals at all chromosomes, enhanced centromeric signals	8277	16973	2.1
Se100C32	CL100Contig32	LTR/*Gypsy*	Dispersed	Dispersed signals at all chromosomes, enhanced centromeric signals	1689	4107	2.4
Se127C69	CL127Contig69	EnSpm/CACTA	Dispersed	Telomeric signals at ∼30 chromosomes	1168	6023	5.2
Se176C39	CL176Contig39	LTR/*Gypsy*	Dispersed	No detectable signal	514	964	1.9
Se164C1	CL164Contig1	Helitron	Dispersed	No detectable signal	665	828	1.2
Se194C1	CL194Contig1	tRNA	Dispersed	No detectable signal	434	824	1.9

*^a^The sequences of listed repeats can be found in Supplementary Dataset 1.*

Eight repeats (LA38C21, LA65C19, LA4C35, LA13C114, LA19C184, LA119C12, LA189C21, and LA143C8) that showed genome-wide dispersed distributions were also selected for FISH (Table 1). These eight repeats were annotated as different TEs and had different copy numbers (340–5,612) in the genome assembly (Table 1). FISH results showed genome-wide dispersed distributions for repeats LA38C21, LA65C19, and LA4C35 (Supplementary Figure 2), consistent with genome alignment results. For LA13C114, other than dispersed signals, stronger signals from centromeres were observed (Figure 2H). For LA19C184, dispersed signals at part of chromosomes were detected (Figure 2I). For LA119C12, spot signals from ten chromosomes were observed (Figure 2J), which is in contrast to the dispersed distributions of the 983 copies in the genome assembly alignment assay (Table 1). Among these ten signals, eight signals showed higher intensities, indicating much higher copy numbers for these eight chromosomes. For repeat LA189C21, spot signals from distal ends of all chromosomes were detected by FISH (Figure 2K). However, the 340 copies identified from the genome assembly showed a genome-wide dispersed distribution. Therefore, these results indicate that LA13C114, LA19C184, LA119C12, and LA189C21 are highly repeated sequences, and their copies may be underestimated or incorrectly anchored in the current genome assembly. In addition, no detectable signal was observed for repeat LA143C8. We found that the 681 copies of LA143C8 showed a widely dispersed distribution in the 80 pseudochromosomes, which might lead to very low signal intensity and low detectability by FISH.

### Genomic Distributions of Repetitive Sequences in *Saccharum spontaneum* SES208

**FIGURE 3:**
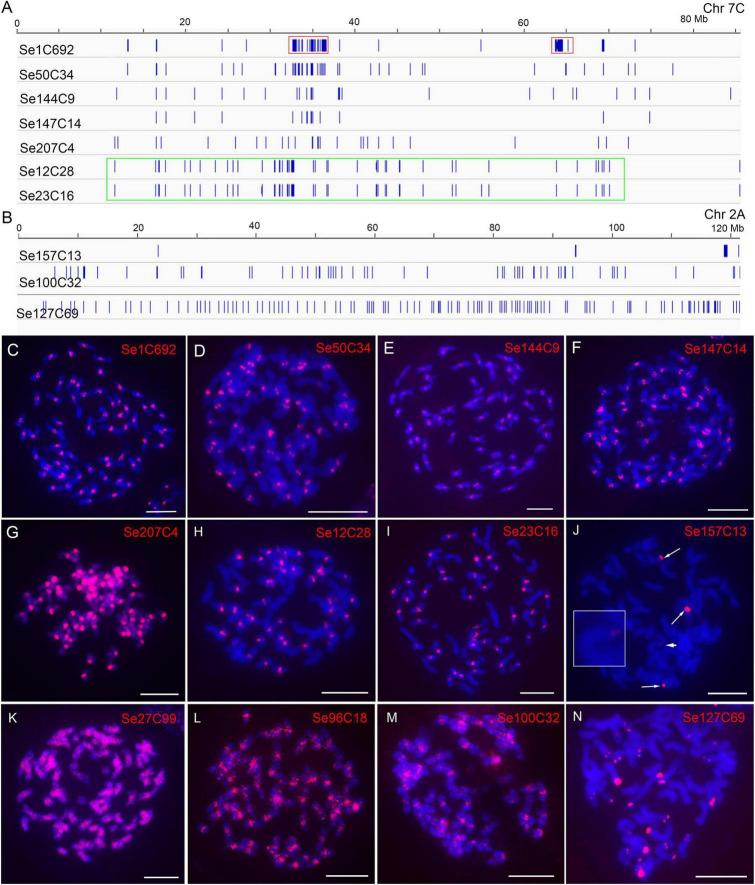
Characterization of genomic distributions of representative repeats in SES208. **(A,B)** Diagrams illustrate the distributions of ten repeats in assembled pseudochromosomes. Red boxes indicate the two separated regions enriched with the copy of repeat Se1C692. The green box indicates the copy enrichment regions of Se12C28 and Se23C16 on chromosome 2A. **(C–N)** FISH analyses of 12 representative repeats in SES208. The four FISH signals from probe Se157C13 are indicated by arrows and arrowheads **(J)**. The chromosome with a weak Se157C13 FISH signal (arrowhead) was enlarged. Bars = 10 μm.

For the 305 repeats of SES208, 218-70086 copies for each repeat of them were identified. We found that repeat Se1C692 had a copy number of 70,086, which was at least four times more than that of all other repeats (218–16,554 copies). Sequence comparison showed that Se1C692 was derived from a centromeric satellite repeat that is the dominant repetitive sequence in *S. spontaneum* (Nagaki et al., 1998; Zhang et al., 2017). There were 227 repeats with a copy number greater than 256 (an average of four copies of each chromosome) (Supplementary Table 2) that were used for subsequent analysis. By genome alignment assay, only 8 among the 227 repeats (3.5%) were found showing non-dispersed distributions (Se1C692, Se50C34, Se144C9, Se147C14, Se207C4, Se12C28, Se23C16, and Se157C13) (Supplementary Table 2). The low proportion of non-dispersed repeat in SES208 is consistent with that in LA Purple (2.1%). Interestingly, the regions enriched with copies from seven repeats (Se1C692, Se50C34, Se144C9, Se147C14, Se207C4, Se12C28, and Se23C16) were largely consistent in the assembled genome (Figure 3A). With FISH, signals from centromeric regions were observed from all seven repeat probes (except Se207C4) (Figures 3C–F,H,I), indicating that they were centromere-specific enrichment sequences. For Se207C4, dispersed signals from the whole genome were detected with enhanced signals in centromeres (Figure 3G). For Se157C13, FISH signals from the distal ends of four chromosomes were detected (Figure 3J, arrows and arrowhead), which is consistent with the computational alignment assay (Figure 3B).

Intriguingly, we observed that the copies of repeat Se1C692 were concentrated in two regions (32–37 and 63–65 Mb), which were separated at an ∼30 Mb distance from each other in pseudochromosome 7C (Figure 3A, red boxes). The obtained results lead to a hypothesized that this was an assembly error in the current genome assembly. To confirm this hypothesis, we conducted dual-probe FISH using the chromosome 7-specific probe Ss7 (Meng et al., 2021) and the Se1C692 probe. FISH results showed clear monospot signals from the centromeres of all eight chr7 homologous chromosomes (Supplementary Figure 3), indicating that the Se1C692 copies were concentrated in a region. Moreover, copy enrichment regions were not found in all pseudochromosomes, which is in contrast to the FISH signals found in all centromeres in SES208 (Figure 3C). Similar cases were also found for repeats Se12C28 and Se23C16. For both repeats, regions enriched with their copies were detected in only ∼10 pseudochromosomes of assembled genome. In addition, the copies of Se12C28 and Se23C16 spanned relatively large regions in most of the assembled pseudochromosomes (Figure 3A, green box). However, we always observed monospot signals from ∼40 centromeres with FISH (Figures 3H,I).

Seven whole-genome dispersed repeats (Se27C99, Se96C18, Se127C69, Se176C39, Se164C1, Se194C1, and Se100C32) were selected for FISH analysis (Table 1). Among them, Se96C18, Se100C32 and Se176C39 were derived from Gypsy retroelements with 8277, 1689, and 514 copies in the assembled genome, respectively. Se27C99, Se127C69, Se164C1, and Se194C1 were derived from repetitive elements of the *Copia*, EnSpm/CACTA, Helitron, and multicopy tRNA genes, respectively (Table 1). Dispersed signals were found from repeats, Se27C99, Se96C18, and Se100C32 with FISH (Figures 3K–M). However, brighter signals from the centromeres were also observed in the Se100C32 and Se96C18 FISH assays (Figures 3L,M), indicating that their copies were highly enriched in the centromeres. For repeat Se127C69, spot signals from only the distal ends of chromosomes were detected (Figure 3N). For the remaining three repeats, Se176C39, Se164C1, and Se194C1, no signals were found with FISH, which may be attributed to a relatively low numbers of copies (514, 665, and 434, respectively) in the SES208 genome (Table 1).

### Copy Number Estimation by Raw Sequencing Read Alignment

As a methodological hurdle to the assembly of highly repetitive sequences, there was an urgent need to increase the number of copies assembled in present assemblies. A copy number survey was carried out based on the coverage depth of raw sequencing reads. Approximately 45 and 40 million reads that accounted for 1 × genome coverage of LA Purple and SES208, respectively, were randomly selected. After mapping to each repeat sequence, the depth of read coverage was obtained to represent copy numbers in the genome (see details in section “Materials and Methods”). The 27 repeats that were analyzed by FISH (Table 1) were selected for copy number estimation by the read mapping method. The data revealed that the copy numbers obtained by read coverage estimation were 2.3–22.0 times greater than those obtained by computational alignment in genome assembly for all 12 LA Purple repeats. Similarly, in SES208, the copy numbers identified by read mapping of the 15 analyzed repeats were 1.2–12.6 times greater than those identified in the genome assembly (Table 1).

### Ribosome DNA Sequences in LA Purple and SES208

**FIGURE 4:**
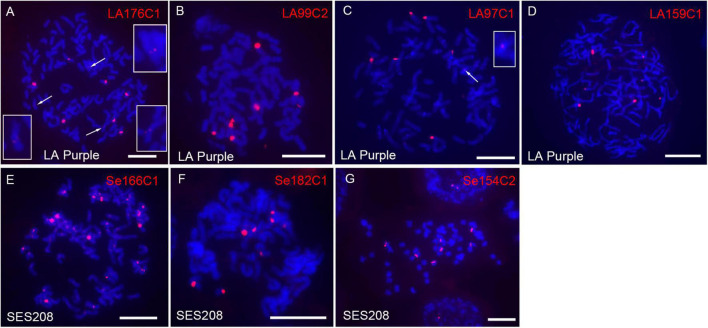
Fluorescence *in situ* hybridization mapping of rDNA in LA Purple and SES208. The seven rDNA-derived repeats LA1176C1 **(A)**, LA99C2 **(B)**, LA97C1 **(C)**, LA159C1 **(D)**, Se166C1 **(E)**, Se182C1 **(F)**, and Se154C2 **(G)** were mapped to the metaphase chromosomes of LA Purple and SES208. The weak signals derived from probes of LA1176C1 **(A)** and LA97C1 **(C)** are indicated (arrows) and enlarged. Bars = 10 μm.

rDNA is composed of highly repeated sequences and is largely concentrated within one or a few regions in genomes. However, the four LA Purple non-dispersed repeats LA1C934, LA137C8, LA103C1, and LA27C11 did not show sequence similarity to rDNA (Table 1). A plausible explanation is that the copies of rDNA sequences were not correctly assembled in the current genome assembly due to the technical barrier in assembling highly repeated sequences. To investigate this hypothesis, we conducted BLASTn with the 190 LA Purple repeats (Supplementary Table 2) as queries. Four repeats, LA176C1, LA99C2, LA97C1, and LA159C1, showed high similarity (>99%, coverage >63%) with the rDNA sequence (Supplementary Table 2). Among these repeats, LA176C1 was derived from 5S rDNA, and LA99C2, LA97C1, and LA159C1 were derived from different parts of 45S rDNA (Supplementary Table 2). However, a total of 409–1520 copies were identified in the genome assembly (Supplementary Table 2), and the copies from each repeat showed a dispersed distribution in some pseudochromosomes in the genome alignment assay. With FISH, ten, eight, seven, and seven spot signals were found for repeats LA176C1, LA99C2, LA97C1, and LA159C1, respectively (Figures 4A–D). Although the signal intensities for each repeat were highly diverse, bright signals were consistently detected for all three rDNA probes. Copy numbers estimated by read mapping showed 2, 7, 18, and 10 times more copies (2438, 8980, 7199, and 5407 copies) than those identified in the current genome assembly for LA99C2, LA97C1, LA176C1, and LA159C1, respectively, indicating that the copy numbers for these highly repeated tandem repeats were underestimated in the genome assembly of LA Purple.

In SES208, three repeats Se166C1, Se182C1, and Se154C2 were annotated as rDNA sequences after searching the NCBI database using 227 repeats (Supplementary Table 2) (sequence similarity > 99%, coverage > 79%). A total of 648, 475, and 839 copies of the repeats Se166C1, Se182C1, and Se154C2 were identified from the genome assembly, respectively, and showed dispersal in several pseudochromosomes (Supplementary Table 2). However, at least 5.8 times more copies were estimated by read mapping strategy (13444, 4337, and 4881 for Se166C1, Se182C1, and Se154C2, respectively). Especially for the 5S rDNA-derived repeat Se166C1, 13,444 copies were identified by read mapping, a number that was 20 times greater than the 648 copies identified in the assembled genome. In FISH, spot signals (∼10 strong signals) from most chromosomes for repeat Se166C1 were observed (Figure 4E). Interestingly, these signals were located in centromeric regions, indicating that the SES208 5S rDNA locus was located close to the centromere, similar to its location in cotton and rice (Koo et al., 2011; Han et al., 2016). However, the FISH result demonstrated that repeat Se166C1 is a highly repeated sequence and was underestimated in the current genome assembly. In addition, FISH assays showed bright spot signals for both Se182C1 (eight signals) and Se154C2 (seven signals) (Figures 4F,G), which is contrary to their dispersed distributions in genome alignment assays. Taken with the above findings, these results revealed that both 5S- and 45S-derived repeated sequences were highly repeated and concentrated in some specific regions rather than dispersed in the LA Purple and SES208 genomes. Moreover, all these rDNA repeats were potentially underestimated in both current genome assemblies.

### Comparative Analysis of Centromere Repeat Distributions Among *Saccharum spontaneum* Clones

**FIGURE 5:**
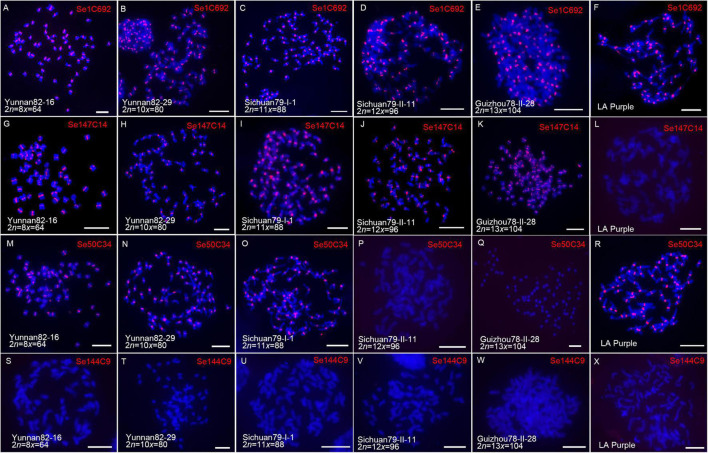
Comparative FISH assay using centromeric retrotransposon-derived repeats in *S*. *spontaneum* clones. Five *S*. *spontaneum* clones, Yunnan82-16, Yunnan82-29, Sichuan79-I-1, Sichuan79-II-11, and Guizhou78-II-28, and *S. officinarum* clone LA Purple, were subjected to FISH. FISH images demonstrated that centromeric signals were detected from the probe of Se1C692 in all studied clones **(A–F)**. For repeat Se147C14 **(G–L)**, centromeric signals were detected from LA Purple and four *S*. *spontaneum* clones Yunnan82-16, Yunnan82-29, Sichuan79-I-1, and Sichuan79-II-11. For repeat Se50C34 **(M–R)**, centromeric signals were detected from LA Purple and three *S*. *spontaneum* clones, Yunnan82-16, Yunnan82-29, and Sichuan79-I-1. Repeat Se144C9 showed no signal in LA Purple and the five studied *S. spontaneum* clones **(S–X)**.

*Saccharum spontaneum* shows the highest level of genetic diversity in the *Saccharum* genus (Panje and Babu, 1960; Irvine, 1999; Mary et al., 2006) with ploidies from 6x to 13x (Meng et al., 2021). In order to study how centromeric repeats evolved along with genomic ploidy change in *S. spontaneum*. Four centromere-related repeats (Se1C692, Se50C34, Se144C9, and Se147C14) located specifically at all centromeres of SES208, were selected for comparative FISH analyses. Five *S. spontaneum* clones with different ploidies (Figure 5), and LA Purple, were used in FISH.

Based on the signal patterns, these repeats can be classified into four types. Repeat Se1C692, which represents the first type, displayed all centromere signals in LA Purple and five *S. spontaneum* clones (Figures 5A–F), indicating that Se1C692 might derive from the common ancestor of *S. spontaneum* and *S. officinarum* and colonize in the centromeres in both species. Sequence analyses demonstrated that Se1C692 shared high sequence similarity (99%) with the centromeric satellite SCEN, a conserved centromeric tandem repeat in sugarcane (Zhang et al., 2017). Repeat Se147C14, representing the second type, showed all centromere signals in the five *S. spontaneum* clones but no detectable signal in *S. officinarum* LA Purple (Figures 5G–L), suggesting that Se147C14 might have occurred or amplified after the divergence of *S. spontaneum* and *S. officinarum.* Repeat Se50C34, representing the third type, showed centromere signals in three of the five *S. spontaneum* clones (Yunnan82-16, Yunnan82-29, and Sichuan79-II-1) (Figures 5M–Q). Intriguingly, we detected all centromere signals from LA Purple in Se50C34 FISH (Figure 5R). Therefore, Se50C34 might arise before the divergence of *S. spontaneum* and *S. officinarum* but show diverse centromere adaptation along with *S. spontaneum* ploidy change. Se144C9 represents the forth type and showed no signal in LA Purple and the five studied *S. spontaneum* clones (Figures 5S–X), indicating the identity of an SES208-specific enriched centromere repeat.

## Discussion

A typical feature of eukaryote genomes is their enrichment in repetitive DNA, which is often greater than the coding sequence component. However, it is always difficult to characterize the exact composition and distribution of highly repeated sequences because of the technical barriers to assemble them. In this study, we adopted a method to assess repetitive DNA composition using similarity-based sequence clustering (Novak et al., 2013). We revealed highly similar compositions of TEs (Figure 1) in the two polyploids *S. spontaneum* and *S. officinarum.* Recent studies based on cytological and genomic comparisons revealed that *S. spontaneum* and *S. officinarum* have a close genetic relationship and may have diverged less than one million years ago (D’Hont, 2005; Zhang et al., 2019; Meng et al., 2021). Therefore, there are two possibilities to explain the high similarity of TE compositions in the two species: TEs in both genomes may have remained steady or TES may have evolved under a relatively similar dynamic after divergence from their common ancestor. The former possibility is most likely because asexual vegetative propagations of both species may have restrained the activities of TEs.

Long terminal repeat retrotransposons are ubiquitous in plant genomes and frequently represent the most abundant repeat families (Kumar and Bennetzen, 1999; Mehrotra and Goyal, 2014). However, the distribution may be diverse for LTR retrotransposons in plants. For example, LTR retrotransposons are enriched in the immediate vicinity of centromeres and relatively scarce on chromosome arms in species with small genomes, such as Arabidopsis and rice (Gao et al., 2004; Peterson-Burch et al., 2004). In plants with large genomes, retrotransposons appear to be abundant throughout the genomes (Jiao et al., 2017; Appels et al., 2018; Sun et al., 2018; Chen et al., 2020). Therefore, we anticipated that LTR retrotransposons made up most of the repetitive sequences and they appeared to be abundant throughout in the respective genomes (Supplementary Tables 1, 2). Direct visualization of retroelement distribution using FISH confirmed that most highly repeated sequences were present throughout the genome. However, our results also showed that each repeat may present a characteristic pattern of enrichment. For example, the Gypsy elements LA13C114 and LA19C184 were enriched in the whole genome and in a portion of chromosomes in LA Purple respectively (Figures 2H,I). In SES208, the two Gypsy elements Se96C18 and Se100C32 showed a whole-genome-wide distribution that was enhanced in centromeric regions (Figures 3L,M). The variation observed in the distribution patterns of different Gypsy elements indicates that the acquisition of new enrichment mechanisms occurred repeatedly during retrotransposon evolution.

Both *S. spontaneum* SES208 (2*n* = 8*x* = 64) and *S. officinarum* LA Purple (2*n* = 8*x* = 80) are considered autopolyploids (Irvine, 1999; Wang et al., 2010; Zhang et al., 2017, 2018, 2019). Interestingly, we frequently observed apparent diversities in either signal locus number or signal intensity for a given non-dispersed repeat with FISH. For example, four telomeric signals with different intensities were observed for repeat Se157C13 in the autooctoploid SES208. Moreover, this occurred in other non-dispersed repeats such as, LA103C1 and LA119C12 in LA Purple (Figure 2). Recent cytogenetic and genome sequencing indicates that SES208 and LA Purple arose from two rounds of whole-genome duplication in a short time (Zhang et al., 2017, 2018). Therefore, it is expected that there were eight signals or a multiple of eight signals from the homologous chromosomes of an autooctoploid and that the signals from one set of homologous chromosomes display uniform brightness if the repeats from homologous subgenomes underwent uniform evolutionary dynamics. In contrast, the diverse FISH signal patterns in SES208 and LA Purple indicate that the homologies from each repeat were subjected to unbalanced amplification or deletion within a short time frame during or after genome polyploidization in sugarcane. In contrast to non-dispersed repeats, no obvious signal diversity from dispersed repeats was found (Figures 2H, 3K and Supplementary Figure 2), indicating diverse proliferation mechanisms for the non-dispersed repeats.

Due to technical barriers, highly repeated sequences are frequently absent in *de novo* genome assembly. Recently, the two polyploid sugarcanes LA Purple and SES208 were sequenced and assembled (Zhang et al., 2018; Ming et al., unpublished), which provides a valuable resource for genetic research and breeding in sugarcane. However, our FISH results displayed discrepancies for some repeats that contrast their distributions in the assembled genome. A feature of this type of repeat is that it is highly repeated in centromeres, telomeric and rDNA regions (Figures 2H–K, 3L–N). A typical example is the rDNAs. Large FISH signals were detected from 7 to 10 chromosomes, indicating highly repeated copies residence. However, only dispersed copies were assembled in current genome assembly, indicating the incapability of current technique to assemble the highly repeated rDNAs. In fact, most of the regions with highly repeated sequences have yet to be finished in current genome assemblies in plants with large genomes. Thus, it is plausible that these highly repeated sequences were underestimated and were not assembled in the current LA Purple and SES208 genome assemblies. Cytological mapping using FISH is a powerful tool to characterize highly repeated sequences with respect to their genome-wide distributions and to finally complete whole-genome sequencing for sugarcane and other species with complex genomes. Furthermore, the application of multiple approaches including raw read assay combining with cytogenetic and phylogenetic analyses (Marti et al., 2021) will be essential for achieving a better understanding for the repetitive sequences in sugarcane or other plants.

A common feature of centromeres is that they are enriched with satellites and TE repeats. Both centromeric satellites and TEs evolve rapidly and can differ greatly, even among closely related species of eukaryotes (Burrack and Berman, 2012; Lermontova et al., 2015). Although centromeric sequences evolve rapidly, these repeats are often homogenized within one genome and thus, a single type of satellite can dominate all centromeres in most higher eukaryotes (Jiang and Birchler, 2013). For example, the centromeres of humans and the model plant *Arabidopsis thaliana* are composed exclusively of the 181-bp alpha (Willard and Waye, 1987; Miga et al., 2020) and pAL1 satellites (Copenhaver et al., 1999), respectively. The process of homogenization was considered a result of adaptation, in which specific centromeric repeat(s) evolved into a structure favorable for the function of centromeres (Gong et al., 2012; Zhang et al., 2014). However, it is still unknown whether adaptive centromeric repeats are steady in centromeres during genome duplication or polyploidization. The species *S. spontaneum* presents an ideal system for the study of centromeric repeat dynamics during genome ploidy changes because there are ∼40 cytotypes (Panje and Babu, 1960; Irvine, 1999; Mary et al., 2006) with ploidies from 6x to 13x (Meng et al., 2021). Our recent study showed that the SES208 centromere is a typical centromere with a dominant satellite and retrotransposon-like DNA (Zhang et al., 2017). As the results showed (Figure 5), we observed consistent bright signals from all centromeres in all studied *S. spontaneum* and LA Purple clones for the centromeric satellite repeat Se1C692. However, highly dynamic signals were found among *S. spontaneum* and LA Purple clones for the studied retrotransposon-derived centromeric repeats. Especially for Se50C34 and Se144C9, the former displayed all centromere signals in 8x, 10x, and 11x clones but not in the higher ploidy clones of 12x and 13x; the latter displayed a SES208-specific centromere localization pattern. These results revealed, for the first time, the rapid turnover of centromeric retrotransposons during genome duplication. In cotton, centromeric retrotransposons were found to spread and proliferate between genomes subsequent to polyploidization (Han et al., 2016). Therefore, a plausible explanation for this finding in sugarcane is that polyploidization triggers the adaptive proliferation for certain retrotransposons. However, we do not know how certain retrotransposons this proliferated and spread in all centromeres in closely related *S. spontaneum* genomes. Due to a limited knowledge of centromere repetitive sequence evolution, uncovering centromeric retrotransposon dynamics along with sugarcane genome duplication will provide new insights into centromeric repeat establishment and targeting.

## Data Availability Statement

The datasets presented in this study can be found in online repositories. The names of the repository/repositories and accession number(s) can be found below: https://ngdc.cncb.ac.cn/, PRJCA007170.

## Author Contributions

KW acquired financial support and provided overall direction of the project. DX, KX, BS, and KW conducted the experiments. KW, HZ, HK, and AE analyzed the data and drafted the manuscript. All authors read and approved the manuscript.

## Conflict of Interest

The authors declare that the research was conducted in the absence of any commercial or financial relationships that could be construed as a potential conflict of interest.

## Publisher’s Note

All claims expressed in this article are solely those of the authors and do not necessarily represent those of their affiliated organizations, or those of the publisher, the editors and the reviewers. Any product that may be evaluated in this article, or claim that may be made by its manufacturer, is not guaranteed or endorsed by the publisher.

## Footnotes

1
https://repeatexplorer-elixir.cerit-sc.cz/galaxy/

## Supplementary Material

Supplementary Figure 1 Summary of the contents of the repeat family and single copy reads in *S. officinarum* LA Purple **(A)** and *S*. *spontaneum* clone SES208 **(B)**.

Supplementary Figure 2 FISH mapping of repeats LA38C21, LA65C19, and LA4C35 in LA Purple. FISH results showed that repeats LA38C21, LA65C19, and LA4C35 were dispersed in the genome of LA Purple. Bars = 10 μm.

Supplementary Figure 3 FISH mapping of repeat Se1C692 and chromosome 7 painting probe in SES208. Probes of chromosome 7 and Se1C692 were hybridized to a somatic metaphase cell of SES208. The merged images from **(A,B)** demonstrated monospot signals from the centromeres of all eight chr7 homologous chromosomes. Individual chromosomes bearing chromosome 7 painting FISH signals are bracketed using dotted lines in **(B)**. Bar = 10 μm.
