# Supplementary figures and images for: Characterization of Repetitive DNA in Saccharum officinarum and Saccharum spontaneum by Genome Sequencing and Cytological Assays
Source: Front Plant Sci. 2022 Feb 22;13:814620. doi: 10.3389/fpls.2022.814620 (PMC8902033; doi:10.3389/fpls.2022.814620)

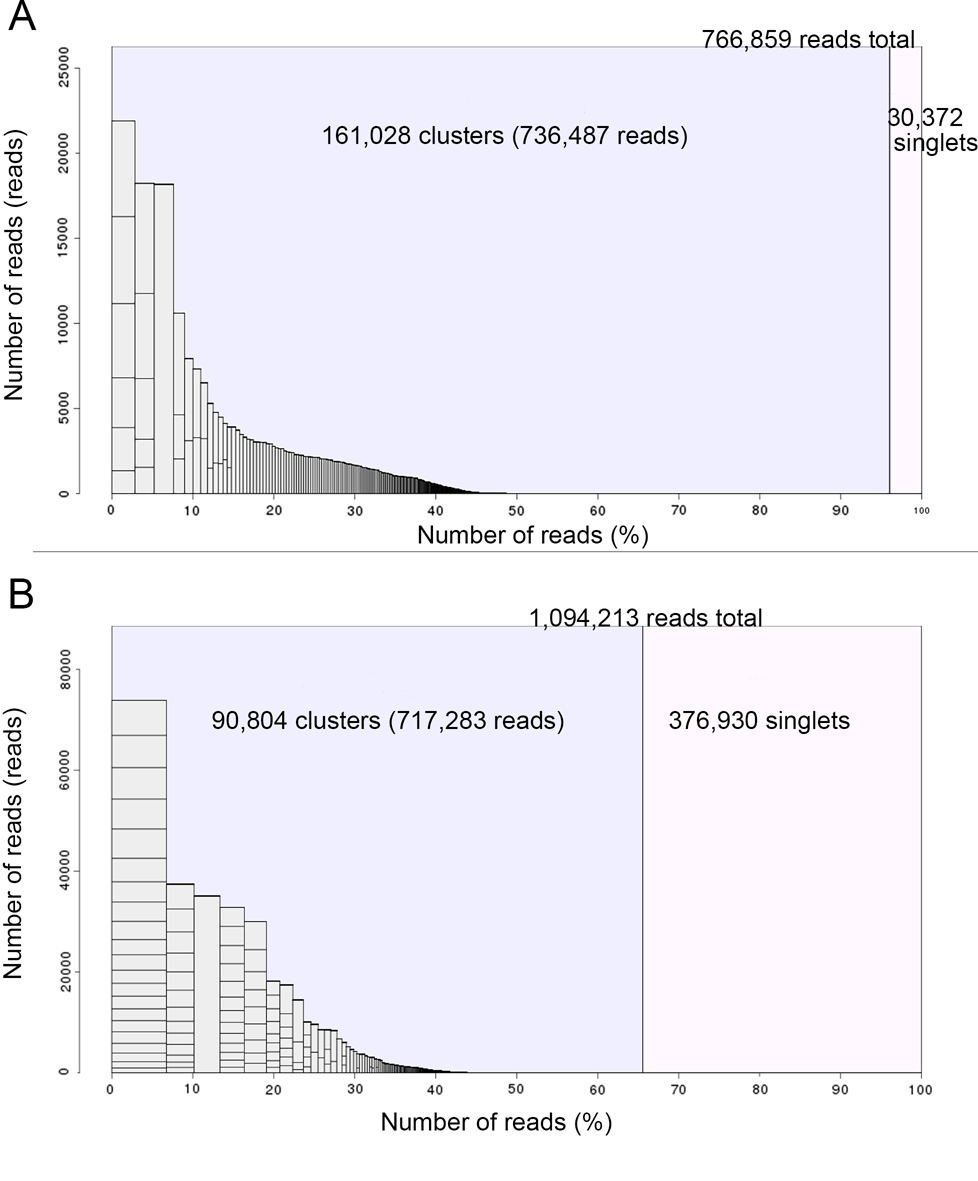

Supplement: Supplementary Figure 1 — Summary of the contents of the repeat family and single copy reads in S. officinarum LA Purple (A) and S. spontaneum clone SES208 (B). [file Image_1.TIF]

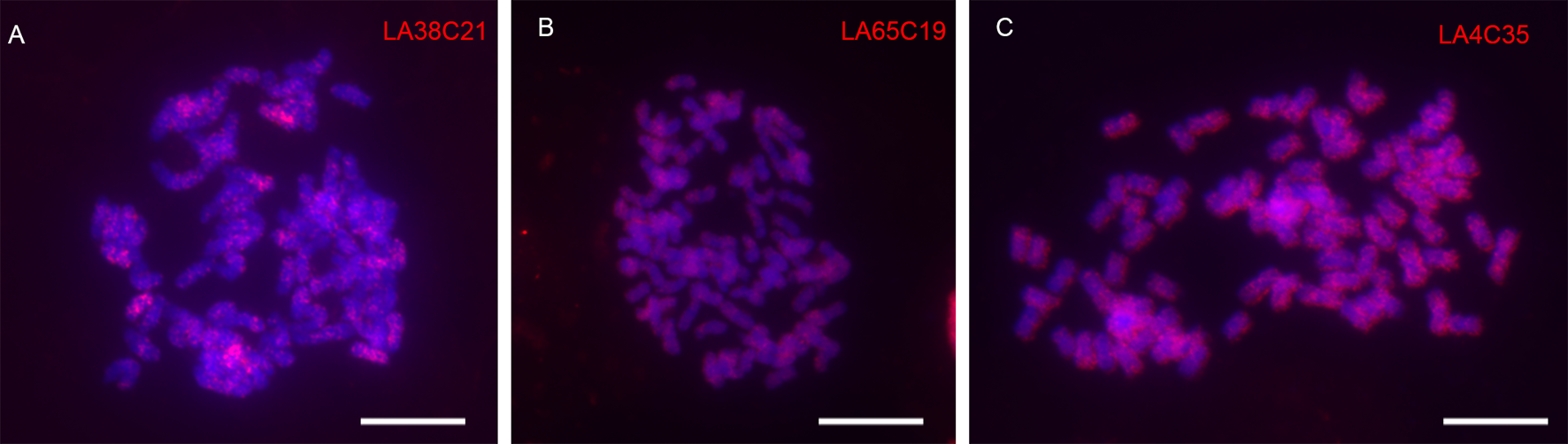

Supplement: Supplementary Figure 2 — FISH mapping of repeats LA38C21, LA65C19, and LA4C35 in LA Purple. FISH results showed that repeats LA38C21, LA65C19, and LA4C35 were dispersed in the genome of LA Purple. Bars = 10 μm. [file Image_2.TIF]

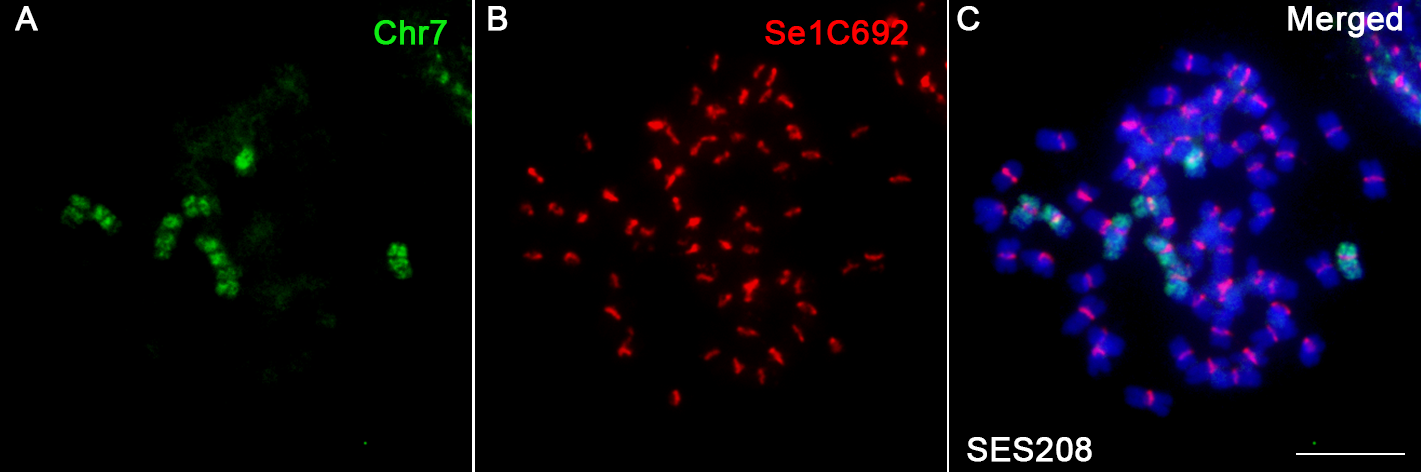

Supplement: Supplementary Figure 3 — FISH mapping of repeat Se1C692 and chromosome 7 painting probe in SES208. Probes of chromosome 7 and Se1C692 were hybridized to a somatic metaphase cell of SES208. The merged images from (A,B) demonstrated monospot signals from the centromeres of all eight chr7 homologous chromosomes. Individual chromosomes bearing chromosome 7 painting FISH signals are bracketed using dotted lines in (B). Bar = 10 μm. [file Image_3.TIF]
