# Supplementary material for: Characterization of Repetitive DNA in Saccharum officinarum and Saccharum spontaneum by Genome Sequencing and Cytological Assays
Source: Front Plant Sci. 2022 Feb 22;13:814620. doi: 10.3389/fpls.2022.814620 (PMC8902033; doi:10.3389/fpls.2022.814620)
Supplement: Supplementary file 4 [file Data_Sheet_1.DOCX]

**Supplementary dataset 1. The sequences of repetitive DNAS in LA Purple and SES208**

**Repeats of LA Purple**

>CL1Contig934

AAGATAGGAGCACAGTTTCCACCGAATATACCATAGGCATAGAAATCGTTTTGGACGCAC

ACGATGGGACTCCTAGGTGACGAGGCTCAAGTGGAAGCTCGGTTCGGTCCGTTTGGAGAT

AGTGCTACTCTTGATGCACGATTGGTGCACGGTTTGCGTCAAACGTACCGTAGGCTCGGA

AATAGTATTGGAGGCACCCGATGGAACTCCTAGTTGACGTGGGTCTTGTGGAATCTCGTT

TCGATCTGTTTTGAGACAGTGTTAGCGTCGGTGCAAGATAGGTGCACGATTTGCGTCAAA

CGTACCATAGGGTCATAAATCAGTTTAGACGCACCCGATGGTACTCCTAAGTGACGTGGG

TCTGGTGGAGCTCTGTTCGGTCCGTTTGGAGATAGTGCTAATCTTGACACAAGATAGATG

CACGGTTTGCACTGAACGTACCATAGGCTCACAAATAGTTTGGGACGCACCCGATGGACT

CGTAAGTGACATGGGTCGTGTGGAATCTCGTTTTAGTCCGTTTGGAGACAGTGTTAGTGT

CAGTGCAAGACAGGTGCACGGTTTGCACCGAACATACCATAGGCATAGAAATCGTTTTGG

ACGCACACGATGGGACTCCTAGGTGACGAGGCTCAAGTGGAAGCTCGGTTCGGTCCGTTT

GGAGATAGTGCTACTCTTGATGCACGATTGGTGCACGGTTTGCGTCGAACGTACCGTAGG

CTCGGAAATAGTATTGGAGGCACCCGATGGAACTCCTAGGTGACGTGGGTCATGTGGAAT

CTCATTTCGATCTGTTTTGAGATAGTGTTAGCGTCGGTGCAAGATAGGTGCTCGGTTTGC

GTCAAACGTACCATAGGCTCATAAATCATTTTAGACGCACCCGAGGTACTCCTAGGTGAC

GAGGGTCTGGTGGAGCTCTGTTCGGTCCGTTTGGAGATAGTGCTAATCTTGACGCAAGAT

AGGTGCACGGTTTGCACTTAATGTACCATAGGCTCATAAATAGTTTTGGACGCACCCGAT

GCAACTCGTAAGTGACGTGGGTCATGTGGAATCTTGTTTTAGTCTGTTTGGAGACAGTGT

TAGTGTCAGTGCAAAGATAGGTGCATGGGTTGCACCGAACATATCGTAGGCATTGAAATC

ATTTTGGACGCACTCGATGGGACTCCTAGGTGACGAGGCTCAACTAGAAGCTCGGTTCGG

ACCGTTTGGAGATAGTGCCACATTTGATGCAAGATTGGTGCATGGTTTGTGTCAAACAT

>CL137Contig8

TCCCAAGCTAAGCAAGGTAGGATTATTCAGATGCTCACACGATTATCGCCCGACTACAAC

TAGGCCACTTGGCCTTTTTTGTTGCGTCTTTGCCAGTGGGTAATAGTGGAGGATGTCCTC

ATCATTCCCACCCCCTTAAACAAGGGCCCAGCTCTGATACCAAATGATACATCCGGCCCA

GGTGGGGTTGGCCGAGGCCCAATAAATTAAGGTTGCCCGGACCCCAACAGTTCTTATGCA

CACCCACTCGGGGAAAGGCCCAATGTCCCTAAAAGACTAGTCCGATAGGGGAAGGGTGGC

TCTCTCTTTTAAGGTGGCTTCCTCCTCCCAAGCTAAGCAAGGTGGGATTATTCAGAGGCT

CACACGGTCGCCGCCCGACTACAACTAGGCCACTTGGCCTTTTTTTGTTGCGTCTTTGCC

AGCGGGTAATAGTGGAGGATGTCCTCATCATTCCCACCCCCTTAAACAAGGGCCCAGCTC

TGATACCAAATGATACATCCGGCCCAGGTGGGGTTGGCCGAGGCCCAATAAATTAAGGTT

GCCCGGACCCCA

>CL1103Contig1

AAGTGCTAGTTCAAAGTTTGTCATAGCTTAGTTCAAGCTCATGATATTTATTTGTCAAGC

TTGTAAGGCTTTCTTGAGCCAAACTATGATCATTCTTTAGACTTGCCAAAGAGTCATTGA

GTGAAGAATGTTCCTTAGCCAAGCGCTCATTAGCATCCTTGGCTAAAGACAATTCTACAC

TCAACTTCTCAACCTTCTCCTTCTCCTTAATGAGCGGTTTTGGGTGATTCGCCGCGACGG

AGTGTCGAAGAATCAACC

>CL127Contig11

AGAAATCCAAAAGCTCTAAGTTTGCACCAAAGGTTGATGAAGGGTTCTTACTTGGTTATG

CTACTAATGAACATGGCTACCGTATTTTCAATGAGGCCACCGGTCAAATTGAAATAACAG

TAGACGTGACTTTTGATGAAAGCGATGGCTCT

>CL38Contig21

TATCATGCTTAATTATTGTTGATTATTATTATGCTCATGTTAATGCTATTCATTATCCTT

ATTACAATTGATCATTGATTCATTAATGGGATTTGGATTACCTTGATGCTACTCAATTGC

TAATTATATGATGATTAAAAGCTAAACGCAGTCAAACAGTGTCTAGCCTTTTTGAGCCTC

ATGAACCCTGTGTTATACTTGTTGAGTTCGACATGGACTCACTCTTGCTATTTCCCCAAC

ACTCCAGTGTGTAGTAAGATGCTACTCGGAAAAAGGACAAAGGTGTAGTGGAGTTTCCAG

AAGACTAGTAGAAGTGCTAGGCATATGTTACCCCCAGTCAGCCGTCCCTGAGAAGAATGG

AGCCCGAAGTTTAGTCACCGTTCCGCGATACTCTGATGTAAGTGTTAATATAGCTATGTA

TACGTTATATAATAACATTGTTTCTGATACTTTTTCATTCTGGCATATGTGTGGACTTCC

TGGGCACACATATGATTAGCCTGGTTTTGTTCTTTAGAAAACCGGGTGTGACAGAGTGGT

ATCAGAGCGGTGTTGACTGTAGGACGTATGCCAAGTAGCACTGAACAATTTCTAAAACTT

ATTTTACGATAAAACTA

>CL65Contig19

AGTGGTACTATTCATCGGGTTGAAATTTTCTTCAATAAGAACTAAGGCCTTGTTCACTTC

CCCATCCCAACCCAAAATATTTCCTCTTCCACCCATCACATCAAATCTTTCGACGCATGC

ATGGAGCATTAAATGTAGGTAAAAAAGATAACTAATTGCATAGTTTGTCTGTAAATGTCG

GGACGAATCTTTTGAGCCTAGTTAGTCCATGATTGGACAAATTTTGCCAAATAACAACGA

AAGATGCTACAGTAACTTTTCACCTACTTTTCGCGAAGTGAACACACCCTAAGTTCATGC

ATACGAGCCATTAGTACAAAAAAATATTCAAGTACAAAAATTATTTGCTACTCACTCC

>CL4Contig35

AAAACTTTTATTATCAAAATCATCTTTGTGAGGGTTGTCATCAATTACCAAAAAGGGGGA

GATTGAAAGTCCCTAGTTTGGTTTTGGCTAATTGATGAAACCTAGGACTAACCTTTGTAT

CTAAGTGGATGATTTAGATAAAGGTGGTCATTGAATATGAGACATGACATTGACTCATGT

GACCAAGATGATGAAGACCAAGATGATGCTTGGCTCGATGGACCGGTTGTGAAGGTGAAG

GGCAAGTCGAGGCTTCGAAGCGAAGGACCATGCGATGGTGAAGCTTGAGCAAGACTTGGC

GCCGACGGACCGATGCAACGGTGAAGAGCAAGTGAAGTCAAGGTCGATGGATCGAGACGG

TCATGTGATGATATGAAGTGGATCATATCATTTGGAGAAACAAAATGGGCTCAAGGCAAA

GGTATAACCATAGGGCCATTTTGTTTTACACTCAAGACACCATAGAGGGTGTGAGTGGTT

TAGGATCGATAGCCGAA

>CL13Contig114

ACTTTGGTATGCTTGCATGATTACTTTATTTATTGCACTTCTATGCATTCATACATATGC

ATTATTGCACCTCATTTAGGTACGCTAGCTGAAGCACGTGAAGGACCTGATGTGGAGCCA

AACCCGAAGATGGAGTTGGTGGATCCATCCCGAAGATAGAAGGACCGAACAAGTGTTAGA

CGGAGGGAATGCTCGCCAAGCGAATTTCATCTGACATACTCTAACTTGAGTCGGATCCCA

GGCAAGCCCCGGAGCATTATAAGCCTCCTATCTTCAAAGCAATTAATTCTATATATGTTA

TAAGTATTGTTGCATTAAGTGGTAGGAGTTGGTTGGAAACCCTTGCTGCATTATATCATT

CCTTGTCCAGATATATGAATCCTTGAATCCTACATAGACTAGGATCGAGTAGATGCTTAG

CCCTGCTTTAAGCGGTAGAAGTCAGGTGATTTCCTGTCACCTGCGAGATATAGGGATATT

CATATATGGCACGGTTGGCTATATTTGCTATCGTGGAATGGAACCATGTGTTAATAAAAT

GAAATGAGACCGGGCGGGATGACGATAGTGAAGCAACAAGGCATGGAGGTCTTGGGTGTG

AATCTATCCCCGTCTGAGTCGTTTAAGGACCGATTCGCTGTACGTCCTTGAGTCATGTTG

AAC

>CL19Contig184

AGGCCTGGAACTATTATGGACGAAAGGGAGGACAAGAATGATTTTGTGGATGATAGATTA

TCTTCTGGCGCTTTTGGCTGCTTGTTGATATTTGTTAACGCACACAGAAAAGGATCCACA

AGCGCACGGATATCGGTGAGCATTTCACCCGGGAGATATTTCCAGAGTATCGTATTTGTT

TATCCGCAAGGAGAAAGCGTGCGATGACTAACCTAAATCTATACTTAACCCCTGGGCCGC

ACTACAAGGTAGCGAAGATATAAGCGATTAGAGAAGATTACGACGATAGTCTGGTTCCAA

CCTATGGTAAGCTATGAAACTATTGGTCTATGGGGACATACCGATGGAAAAGAGACACCA

CAGAGGATGCTTGACTAGGGCACTTAATCCTACCAATCCTTCTAACGGGATGTGGGGTAC

AAAGGAAGCAACGGGATTGTCACGTCCCGCGCTACTACCACGGATCCGGCCAGACAGGGG

ATATCTACGAGTATCTCTAGCCTAAGCACCACGCTTACGCTACAGACGATTACCCTACCC

TCTACCATGGAAGAGAATAAAGTAAACTCGTAAACCATAGAACAATAATAACAACGTACT

TACTTAGTATTAGAAGTTGAATTACCGAAGAATCCTAGAACCAAGCCTCGGGTTAGGAGA

CTTGATCCCGCAGGTACAAGCCGGAGAGGAGACACCGACATGCCGGGCTTCCTCCAACTC

ATCCCTCCACTCTAACTCTCTCAATCTAGAAGAAGAACTCTAATCTCACATGGAGACTAC

AAAGCTAATCTCTCTTGTATGTGCCCTAGGAGAGAAGATCCATTCAAGGGACCCCTCTCA

GCTGATCCCAACTAGAAATAATGAATTAGGGTTTCATGGGATTGCTCCTCCAGGGTCCAG

GGGCCTTGCTTATATAGCCCCCTAGGATGCACGACAGCCCTTGGATCAAACCGACTTTAA

TCAACGGTGATTATTGATCCTCAAAGGCGGTGGAGGCATGATCCGCGAGACTTGACCTGA

TTGGTCCACGTAGGTGGGCGGGCGCCCAAGAAGGTAGGGCGGGCGCCCTGCCACCATGGC

AGATCGGCCTCCCGTTCGTTCCGGTGCCTTCTCGTGTCTTCTAGAGTCTTCCTTCACGTG

CGTGGGTCTTT

>CL119Contig12

ATGCACATGCATAGTGTTTGCATAGTGCATGCATTAACGCCCTGTTTAGTTTCATCAAAA

TTGCGAAAAAAATTCAGAAAAATAAAGAAAAAATTGTCAAAGTTTTTGCACGCTTTTCAG

AGAACGTGCTGTATTTCTGTAGACCACATCCCACCTCAGTTGTAGGTGCCAATTTTTGTG

GTTGCATCTTTTTTGATCCTTGTTCAGAGAAAATTATAAAAAAATAGTGAAAAAATAAAA

AAAGTTTGATTCCTACTAGTGCCTTTTGGAAAAATCAGGGAAAAATTCAGGAAAAAGGAA

AAATCCGTGTTATTTGCTTGTTCTGTGCGTTCTTTCCATCGTCCTTTGTTTTCACCTTGT

TGCGCTACGTCTCGACACGTCTTAGCCAGCAGCTGTGATTTGTACTTTGGATCCGGATTG

AACAATCGCTAATCTGCACTCGTTAATTGCTAATCCTTGCTGTCATATTGTGTGCCTACC

CATAGCTCCACATATTCTTCTATCAGCACAGGTTCATCTTGCAACTGTTACCCACGCTCA

ACAACAGATTGCTTCGCCCTGTTTGGTTTCATCGAAATTGCCAAAAAAATTTCAGGAAAA

TAAAGAAAAAAAATTGTCAAAGTTTTTGCACGCTTTTTAGAGAACGTGCTGTATTTCTGT

AGACCACATCCCACCTCAGTTGTAGGTGCCAATTTTTGTAGTTGCATCTTTTTTTGATCC

TTGTTCAGAGAAAATTATAAAAAAAATAGTGAAAAAATTAAAAAGTTTGATTCCTACTAG

TGCCTTTTGGAAAAATCAGGGAAAAATTCAGGAAAAAGGAAAAATCCGTGTTATTTGCTT

GTTCTGTGCGTTCTTTCCATCGTCCTTTGTTTTCACCTTGTTGCGCTACGTCTCGACACG

TCTTAGCCAGCAGCTGTGATTTGTACTTTGGATCCGGATTGAACAATTGCTACTCTGCAT

TCATTAATTGCTAATCCTT

>CL189Contig21

TAAAAGTTGTCAACAACGAAGTTTCATAAATTTTAGGGATCTACAACTTTCATTTTGGTA

GTTTCTATGTTCGAGGTAATTTAGGACCTGTCATACACCGACCGGCCTATTTGCACGATA

TGGCCCACAAGACGATTTCAAATCAAAAAGTTGTCAACTACGAAGTCGCGACACTTTTCG

CGCGAAAATCATGCGCTTTGCGGCTGCGAGGATTCGAACCCGCGGCCTCCCCTCGCGCGT

AGCCTCCTCTATCACTGCACCTCGTGCTCACATGTGCCTATAATGGATTTTGGTTCCCCA

CATATTATACTAAACTGAGCATAAATTGATTGTTTGACGCCCTAAACGATTTCAAATGAA

AAAGTTGTCAACTGCAAAGTTTCATAACTTTTCGAGATCTACAACTTTCATTTTGGTAGT

TTCTCCATCCGAGGTCATTTAGGACCTGTCGGCCAGTGGGCGGCCGACAGGCCTATTTGC

CCGATATGGCCCACAAGATGATTTCAAATCAAAAAGTCGTCAACTACGAAGTTTCATAAA

TTTTTGGGATCTACAACTTTCATTTTGGTAGTTTCTCCGTCTGAGGTCATTTAGGACCTG

TCGGCCACCGACCGGCCTATTCGCCCGATACGGCCCA

>CL143Contig8

TACTTACTAGTTAGTAGTATTTTTGTATTTACTTTTTGTGTAGATTGTAGTAAAGCAATT

GTGCTTCGTTATGTTTGGGAACTAGATGGACAACCTAGTGAGCATATATCATGGAGGAAC

TGTGGAACGAGATCGCTATGGATATGTTGAGTTTGTTGACATGCAAAGTGTGCCTGTGCT

ATTCAATGAGAAGCCATCATTTAGTGAGTTGACTGCAAGGGCTCGTGAGGAGGTACATTG

CTATGGAGATGATGGCATCATAGTTGAGGGTGTACTTCACCTAGGTTTCCCTCCCAACAT

GCTAAGGAAGATGATCCCAATAGGGTGTGCAGATCAGTGGGACAACTATGTGAGATCGGC

TATGAAAAGCCAGTTCCAAAGTTTGGACGTGGTTGTGCATCGGGTGCTCGTTGATCCCAT

CCCTCTTGGGTTGTCCCCGCCAATGGGTGAGCAGGCATACTTTGAACCTCCCGTCCCGGA

ACGTGATGTGGATGCGGAGGTTCCGCCTACCGTTCCCGATGCGCAATCTGCCCCCAATGA

TGTGGCACATCCTCCTCAGGAGATCCCTTTGACACAGAATCATCCGAGTAAGTGTCAATT

AGGCATCGTTTTATCTTTGTAGCTTAAGTCTCTTCTTTTCATCCACTTTTCTTATCATCT

CCTCTTATTTGTGGCTATGTTGTAGGAGACGTTCCCGATAATGAGGGCGCTCCTCCTGCT

GATGCTCAAGTCCAGATTGGAGATGGAATCCGTGCCTCCAATAGTGTTGAAATTATGAAT

GATTCAGAGCCATATGAGATCGCAAGGGCTGTTGATTCTGACGATGATCGTCCCGTTGGA

GAGCTCACGGAGAGTGACGTTGAGATGCTGAGGCGTATTTTTCCCGGCCGCCGTGATCCT

ATGGTTCATGAGTTCAGTGATCTTACTCATTCAGATCAGGCGTGTGCCGAAGGACGTGAT

GATGAGCTGCAGGAAGCTCCAGAGGCTGGTCCTAGTATGGAGATAGAGAAGGGGAGGGTT

TTCAATGACCTCCCTGCATTGAAGAGGTGGTTGCAGGCGTTTGCAGTGATACGCAAGAGA

CCGTACAAGGTCCTGCATTCCTATGTGGAGCGGCGTTACACGGTTGTGTGTGACAAGGAA

CGCTGTCCATGGAGGGTTTGTGCAAGGAAGCAAAAGGTCTCAGGAAAGTGGAAGATCACA

AGAGTTGTTGGGCCGCATACCTGTTCCTCACATGACCTGAACACAAAGCATCGGCAGTTG

ACATCAACCCTCATTGCCAAGCGGTTGATGAAAGTATTACAGGGAGAGCCGAATATGAAG

GTCAGGACAATTATCAAAATAGTTAATGAGGTATATGGAGGATATGACATAACTTATGGC

AAAGCCTGGAGGGCTAAGCAGCGGGCGTGGAAGATGATATATGGGGACTGGGAGGATGGC

TACGAGCAGCTTCCAGTACTTCTCAATGCAATCAAAGCAGTGAATCCAGGCATGCATTAT

GAGTACATCCCTAGGCCAGACGCATGGAAGGATGGGAGACAGATATTCTTCCGTGCTTTC

TGGTGCTTCCCTCAGTGTGTCGAGGCCTTTAGGCACTGCCGTCCAGTCTTCTCCATTGAT

GGTACATTCTTGATTGGCAAGTACCGTGGCACACTTCTCATAGCCATATCGTGTGACGCA

AACAACAAGCTTGTTCCTTTGGCATTTGCTTTGGTTGAGAGGGAGAATAATGATAGCTGG

GGATGGTTCTTGAGGCTAGTCCGGATACATGTGGTTGGGCCTGGCAGGGAGGTGGGCGTC

ATATCTGATAGGCACCAGGGCATACTTAATGCTGTGCGAGAGGAGATAGAGGGGTATGCA

CCTTTGCACCATCGTTGGTGTACTCGGCACCTTGCTGAGAATCTACTCCGGAAGGATGGT

GTGAAGGATAATTTTGATCTTTTCCAGGAGGCTGCTCGACAGCTTGAGGATAGGTATTTC

CGGAGGAAGTTAGAGGAGGTCAGAACCGCATCTAATGCAGAAGGTAGACAATGGCTGACA

GGTTTGATGAGAGATGTGGAGAAATGGACAAGAGCTCACGACGCAGGTGGTTGGAGGTAC

GAGTTTCAGTGCAGCAACATGGCCGAGTCTTTTAATAAGCTGCTGCTGGGGATTCGTGGT

ATGCCCGTGAACGCTATCGTTCAATTCACATTCTACAAGCTTGTTGCCTGGTTCAACGAT

CGGCACGCCCATGCATTGCAGTTGCAGAGTGAAGGTAAGATTTGGGCTCCCACACCACAT

GCACATCTAGACAAGGCGAAGGAAAGGGCTGGCACACATGAGGTCACATGTTTTGACCAT

GCCACAGGAAGATATGAGGTGAAGCATACCGGTGGTACTACATCTGATGGTGAGGTCCGA

GAGTCGAGGATTCATGTGGTAGTCCTCCAAGATTTCTCATGCACTTGTGGGAAACCAAGG

CAGTATCACTTTGTATGTTCTCACCTTGTAGCAGCCGCTAGGCATCGCAACT

**Repeats of SES208**

>CL1Contig692

ATCTCATTTCGAACCGTTTGGAAATAGTGTTAGCATCGGTGCATGGTTTGCGCCAAACGT

ACCATAGGCTCAGAAATCATTTTGACGCACCCGATGGTACTCCTAGGGGACGAGGCTCAA

GTGGATGCTTGTTTTGGTCCGTTTGCAGATAGTGCTAACCTTGACGCAAGATAGGTGCAT

GGTTTGCGTCGAACGTACCATACGCTCAGAAATCGTTGTTGGACGCACCCGATGGATCCC

CTAGGTGACGTTGGTCATGTGGAATGTTGTTTCAGTCCATTTGGAGATAGTGTTAGTCTC

AGTGCAAGATAGGTGCACGGTTTGCGCCAAACGTACCATAGGCTCAGAAATCATTTTGGA

CGCACCCGATGGTACTCCTAGGTGACGAGGCTCAAGTGGATGCTCGTTTTGGTCTGTTTG

GAGATAGTGCTAATCTTGACGCAAGATAGATGCATGGTTTCGCAGAACGTACCATAGGCT

CAGAAATCGTTTTGGACGCACCCGATGGTACTCCTAGGTGACGAGGCTCAAGTGGATGAT

CGTTTCGGTCTATTTGGAGATAGTGCTAACTTGATGCAAAATAGGTGCACGGTTTGCGTC

GAACGTACCATAGGCTCAAAAATCATTTTGGACACACCCAATAGAACACCTAGG

>CL50Contig34

ATATGAGGCTTATCATTGATGCATTTGATGAATTGGTGCTCCACCATAAAATGTGTATAT

TTTCATTTTCAGAATTACTTACATGGATCAAGGAAAGGTTTGATTGCATATGGAAAGCAT

GGAAGGTTAATGAAGTTGATTGGGGACCAAATCCAAGTATTCCCAACGTCCTCCACCTGG

ACACCACGTCACTTTTGGTCCAAGGAAAGAAAGAGATCAAATCCAACACGTTTTGGGGCT

GGATTCGGACTCCAGGACAGCTCCAACTTGTGATGTTCACCACGGTCGCATACGGACTCG

GATTGGGACGTTCTAGCACTTTTTGGAAAGCTTATCAAGTCTATTTTCCAACGGATCTGG

ACTCATGGCTATATCTTATCCGATGCTTCCGCAATCGTCGTTTCAACGCCGGGGCCTTTT

CTGCCTTTGGTGCTGTGTCACCCTATTTTGGGCTGATGGCCCATGTATCAAGTTGGGTTC

ATTAGGGACGTGTCCTAGGGTTGGAGAACGACCCCAACACCCCCTGGTCGTTCCCCTAGG

TATTAATAAGGATTAGAGCCGCCACAAACAGATTGGGTTTTGTTTTGTTGAAAGTTTAGC

CATTGCTACTTCCTTGTAGACGCGTGTGTCGACTAGACCATCCGTTCTACTCGATTCAGA

ACCCCAACTTTGTGATTTCAGATTGGTTTTCATCTCCATATTTGCAATTGAGTTGCTTGT

TCTACTTGTTCTTGCTTGTTCCTCGATTGCTTGCAGGAATTACCCTAGTGGTTTGGTTGA

TCGTGTTCCACAAGATCGCGACGGCTGTTGGAGGTGGTGTATCGGTTGCTAAGGCGCAGC

ATCCTTGGATGGTTGTAGTCGGGCCGTGAACGTCATCTCCATCCCCAAATCGAGTTATCC

ACCTTCTCTCATCGAAAGATCAGGATTCACCCTAGCGGGTTCATATCAGTTGGTAATCAG

AGCAAGGTTGATCGGTGAGAGACTTCTAGTTCTTTGCTGTTTTTAATCTCCTA

>CL144Contig9

GTTTTGTTTGGTTGTTTCATGCAGTTTCAACATCAATTCAGCACGTCGCTTAGCATCAAA

ATTTAACTTTTCTGAAGTTGGTAAAGGCATTAAATCAATAGGAGCACGTGGCAACAAACC

ATAAACAATCTCAAATGGACACATCTTTGTAGTAGAATGCAGCGAACGATTATAAGCAAA

CTCAATATGAGGCAAACAATCTTCCCACATCTTAATATTCTTCTTTAAAACAGTCCTTAA

CATAGTAGACAAAGTTCTATTCACAACTTCAGTTTGACCATCAGTTTGGGGGTGACAAGT

GGTGGAAAATAAAAGCTTAGTCCCCAATTTGGCCCATAAAGTTCTCCAAAAATGGCTAAG

AAATTTAGCATCACGATCAGAAACAATTGTATTTGGCACACCATGCAAGCGAACAACTTC

TCGAAAGAACAAATCAGCAATATTTGTAGCATCATCAGTTTTATGACATGGTATGAAATG

TGCCATTTTAGAGAATCGATCCACAACCACAAAAACACTATCTCGCCCTTTCCTAGTCCG

TGGCAATCCCAACACAAAATCCATAGAAATATCCTCCCAAGGAGCACTAGGAACAGGAAG

AGGCATATACAAACCGTGTGGATTCAACCGGGACTTAGCCTTTTGACATGTTGTGCAGCG

AGCAACAAATCTCTCCACGTCTCGTCGCATCTTTGGCCAAAAGAAATGACCAGCAAGAAT

GTCCTCCGTCTTCTTCGCTCCAAAATGCCCCATCAAGCCACCTCCATGCGCTTCCTGCAG

CAACAACAAGCGAACGGAGCTAGCTGGAATGCATAGCTTGTTAGCTCTAAACACAAACCC

ATCATTGAGGATGAATTTGTTCCATGTTTTCCCATCTTTACAATGCAGCAGCACTTCTTT

AAAATCAGCATCATGAGCATATCGGTCTTTAATTGTTTCTAACCCAAAGATCTTGTAATC

AAGTTGATTCAGCAAAGTATATCTCCTAGACAATGCATCTGCAATGATATTATCCTTCCC

TTTCTTGTGTTTGATAACATAAGGAAAAGATTCAATAAATTCAACCCATTTTGCATGTCT

ACGATTCAGTTTTCCTTGACTACGAATATGCTTCAAAGATTCATGATCAGAATGTATAAC

AAATTCTTTGGGCCACAAATAATGCTGCCATGTTTCTAATGTCCGCACTAGAGCATATAA

TTCCTTATCATATGTAGAATAATTTAGAACAGGCCCACTCA

>CL147Contig14

GTATGCATCAGGGTCATATTTACCATCAAAAGGAGGTATTTTAAATTTAATCTTACTGAA

AGCATCATCATTATTGTGTACCTCGCGTCGGCGGCGGCCACCCATACCTCTACGGTTGTG

ACGTAGGCGACGTCGATCACGAGTGTCTCGATCATCATGTTCAGTATCACCTGTGTATTC

TTCATCATAACTACCATCCTCTCGATCTTCCTCCTTCTTTTCTTCTTTATGCTGGTCTTT

ATCATTGTTGTGGAGGTCATCAAAACGCCTCAGCAGAGCAGCAAGGCTTTTGTCCACACT

AGCAATGGACTGCTCCAAACCTGCAAGCTTGTTGTTTGCGGCAATCTGAGTAGCCTCCAA

CTGCCCAAGTTTGTCATTTGTCACCTGCAAGTCATTATCAAGTCCCTCCGTGTGCAACTT

CACTTTCCTTTCAAAATGTTGTATGATGCCCTTTGTGCGAGGCGAATGTGGAATTTGGTT

ATCTTCGTCGGTCCCTGACATGGTTCAAAGACAAAGACAACAAGAAGACAAGTGAAGAAA

TAAAAGCCCTACAACTACTAGGATGTAGCTACAGCAAGTCGCTCACTCTCCACCTATTAC

ACAAGTTCTTACCAGTTCTTACCTTGCTCGACAGGAGGGGTCATCTACCAACAAGTCTGC

AGCAATGGATGAAGTGTATCGGTGCCGCAGCACGAGACCTGTCAAGCTGTAGAATATGTG

GAGCTATAGGTGGGCTGAAACAAGGAACGAACTAGCACCACGTTAGTTACAAAAAGCAAG

CTGAATAATCGTTCAACCGGCGGTACTGTGCTGGTCCTAGTCTAGACCGTGCTAGAGACG

CGAGCCTGGACACAAAGGAGAGCACAACACACCACCCAAAGTGAACGAAGAATCTCTAAG

ATCAGCCCCTTTCTCTTCTTTTCTTTTGCTTCTTTCTTTTTCTTTTTTTTCTTTTT

>CL207Contig4

GGTATCAGAGCTGGGCCCTTGTTTAAGGGGGTGGGAATGATGAGGACATCCTCCACTATT

ACCCGCTGGCAAAGACGCAACAAAAAAAGGCCAAGTGGCCTAGTTGTAGTCGGGCGGCGA

CCGTGTGAGCCTCTGAATAATCCCACCTTGCTTAGCTTGGGAGGAGGAAGCCACCTTAAA

AGAGAGAGCCACCCTTCCCCTATCGGACTAGTCTTTTAGGGACATTGGGCCTTTCCCCGA

GTGGGTGTGCATAAGAACTGTTGGGGTCCGGGCAACCTTAATTTATTGGGCCTCGGCCAA

CCCCACCTGGGCCGGATGTATCATTTGGTATCAGAGCTGGGCCCTTGTTTAAGGGGGTGG

GAATGATGAGGACATCCTCCACTATTACCCGCTGGCAAAGACGCAACAAAAAAGGCCAAG

TGGCCTAGTTGTAGTCGGGCGGCGACCGTGTGAGCCTCTGAATAATCCCACCTTCCTTAG

>CL12Contig28

TGGTTGCATCTTTTGTGATCCTTGTTCAGAGAAAAATATAAAAAAAATAGTGAAAAAATA

AAAAAGTTTGATTCCTACTAGTGCCTTTTGGGAAAATCAGGGAAAAATTCAGGAAAAAGG

AAAAATTCGGTTATTTGCTTGTTCTGTGCGTTCTTTCCATCATCCTTTGTTTTCACCTTG

TTGCGCTACGTCTCGACACGTCTTAGCCAGCAGCTGTGATTTGTACTTTGGATCCGGATT

GAACAATCGCTAATCTGCTTTCGTTAATTGCTAATCCTTGCTGTCATATTGTGTGCCTAC

CCATAGCTCCACATATTCTTCTGTCAGCACAGGTTCATCTT

>CL23Contig16

TCCCTGATTTTCCCAAAAGGCACTAGTAGGAATCAAACTTTTTTATTTTTTCACTATTTT

TTTTATATTTTTCTCTGAACAAGGATCACAAAAGATGCAACCACAAAAATTGGCACCTAC

AACTGAGGTGGGATGTGGTCTACAGAAAAATAGCACGTTCTCTGAAAAGCGTGCAAAAGC

TTTGATAATTTTTTTTCTTTATTTTCCTGATTTTTTTGGCAATTTTGACGAAACCAAACA

GGGCGAAGCAATCTGTTGTTAAGCGTGGGTAACAGTTGCAAGATGAACCTGTGCTGACAG

AAGAATATGTGGAGCTATGGGTAGGCACACAATATGACAGCAAGGATTAGCAATTATCGA

AAGCAGATTAGCGATTGTTCAATTCCGATCC

>CL157Contig13

TGCAAAAGTGGAACACCAAAGATTTTTCTAAAAATCATTTATCATAGAACACAAAGTAGC

ATTTGTTTCGGTGAAATCTTGCAATTCCGTCGAAGATGTCTGGGTCCACCCGTGTTCCGA

AACAAGCTGGGTAGGACAGGAATGGCACATAGAAAGTTAGGGTGGATAGGTTAGATGATG

TCACTATCAATTACATCACTTACAT

>CL27Contig99

GAGGTGACCGGACGCGCCGGTCGCTCGTAACGGTCGAAATTCTGAAACTTCGGCTGAGTG

ACCGGACGCGCCGGTCAGGGGTGACCGGACGCGTCCGATCGTGCAGGGAGCTTACTGTAA

CGAGACCGGACGCGCGACCGCGTCCGATCGGTGATGACCGGACGCGTCCGGTCGTCGCAG

GACCTTACTGGAACGTGACCGGACGCGCCGATCACACCCAGGTCGCGTCCGACGGGTGTT

TCCCCTCAAGTGACACGTGTCAGCCACTGACCACCGCGTCCGGTCCTCGCGAGGACCGCG

TCCGGTCGTCAGGGCCGAAACCCTAGCCGCGGTAACGTACTGACCGGACGCGTCCGGTCA

TGCCGAAGACCGCGTCCGGTCAGTGGAAAACAGCCCGAAACTTTCCGAAACGCGTCGCAA

CTTCAACCAAGTTGCTTCCAACTGAACTTTGAGCTATTCTAGGGTTGCCTAGCGCCAAGT

TTAACAAGTGTGCACCACACCTAAACATAGGACTCGCCTAGGTCAAGCTACCCGTTCATG

CCCCCCTTTATAGTACGGCCAAAAGAGAAAAACGAAGATCCTAACGACTATTCCGAATGT

ATCTCAACACCTCGACATCCGGAACTAACCGTCCTTAATCTTGACGTCACTCTTTTGAAA

ACCGAAACGATTTCCATCAAGGGACACGAAGCCACAGTGCTCAAAGCAGTTATCACCTCC

ATGACCTAACTTACCAAAGTTTCTGCAAAACACACGTTAGTCATGGTAATAAAGCGTTGT

CATCAATTGCCGAAACCAAACCTTGGGGCCTAGATGCTTTCAATCTCCCCCTTTTTGGTG

ATTGATGACAACACTAAGTTTCAAAGGATTAGAGTGAAGGTTTTCGAGCATGCTTGGTTC

ACATAAGCAAAAGACAATAAGCATAAAAGGTTAGGAATTGCTTATGTGCTCCAAGCCAAA

AGAGTGTACTTCATAAGATAAGACAAAAAGCGTTAGTACACATAAAGATATAGC

>CL96Contig18

AACCAAGCACAACTTGATTGACCTCGCCAAGAAGGCTTTTCCTGCAAGCGAATCGAAGAA

CACAAGCAAGAAGGTAAAACACGCAATCTGAAATTGCAAATATGAATGATGCGAATATCA

ATAGAGGGTTCAAGAACTCGGTTCCAAAGGACTAATCGACACAGTGGAGGAGATCAAGAA

CAGGGGCCCTGGATCACTGTAAAAGGATTTGTCACCACAGTTACAATGAACGATTCAGTT

TCTCGATGGAAAACTAAACTCTAAACAAAACCCAATTGTCTAGCAGCGGCGGCGGCTGTG

TTTATAGTCTAAGACTCGACCTAGGGTTGAGGACGGCCAGGGGTTGGGCGCCCACAACTT

GGGCTTAAGGCCCGACACGATACATGGCCAAGTTGGCCCAAATAGGTGACGCAGCACCTT

GCCGTGGTCACACAGAATGATCCACGGACCTTCTGGAGCTGGGACCAGATCCAAAACGAC

GGCGTCGTCGTCCCCTTTCCAACGCATCCAAGAACGCCCCGTTTCGATGTCGTATGAGAG

AGTTATGACCGAAACAGTGACGACGTGTCTGCTGAATCCGAGGGTGACGTGGCAGCTGAG

TTGGGGACGAATTGCAACTTGGGGAAGACCATAGCGTCGGTGTGTCCAGCGTGGCGATGT

CCTCATCATCCTCCCCTTCTTGAATTGGAGTCGTCCTCGACTCCATCTTGGCGATGTCCT

CATCATCCCCTCCTTCTAGAATTGGAGTCGTCCTCGACTTCATCCCATCATCAGCGAACT

CCTGCAAAAGGTTAGTGACAGCAGGCAATGCACCAGACATAATAGGTTGACAAAACATAG

TATAACATGGTGCATCAGGATTATCACATAACTCAGCAGTAGCAGAAGTTGTAGCAAGAA

AAGTACCTCCTTTTAACTTAATCCCATTATTTTTAGCAATGTCAGTTGGAGGTTTATTTT

TAATCTCTTTAGCATGTTTAATAATATCCGTAGGAGAC

>CL100Contig32

CAAGCAACATCGACGCCGAGGACGGCTTTTCATCAAGAAGGGGAGGATGATGAGGACATG

GCCTCCACACATATGACCATGCTTGGAGAATTACATGGAGGCCAAGGAGATCAGCAAGGG

CATCCAAATCGAGAAGAAGGCCCAAAGCTTATCCGGTTCGAGTCACCAAGGTGGAGGCCC

AAATCAAGTTCGAGTCCACCTCGGAATCCAGGACCAGTCTGCCTTGAACTGGTCACCCAG

GACGCGTCCGGACTCCGTTTTCGATGATCCATATATGGATGGAAAGCTTATTTGATAAGG

AAGCCAACCCAAGTGGTCCCACGTCAAAAGCTATTCGGAATCAACGGGAATCGTCGAAAC

AAGTCACCGTCCAGAATCTGTCAGGGCGCTGTGTCGCCGTCTTTTGGGCCGTTTGGGCCT

TGTAACGTGTCGGCACTCCCCAGGGACGCGAATAGGGGTCCTTGGACGTCCCTTAGGCTA

TATAATAAGTAGTCGCCGCCTACATTAGGGTTGGGTTTTGCTTAGATTATTCTGTTGATA

ACAGTTTCGCCGCCGCCGTCGGTTTGTGAGACCCCAACTTTGTGAGATTAATCATTCATC

TGCAATTTGGTTGCATTCCTTTGTGTTCTTGCGTGTGTTCTCGATTCGCAGGCAAGGACT

TAGCCTTCTTGGCGAGGTCGACCGTGCAACGCCGGTCGATAACCAGAGGAGTCGTGGTGC

TGCGATTGCGGGGTTCAGAACCGAGTTGTTCGGAAGCCGGATCG

>CL127Contig69

ATCTCGAAAAGTTATGAAACTTTGCAGTTGACAACTTTTTGATTTGAAATCATCTTGTGG

GCCATATCGGGCAAATAGGCCTGTCGGCCGCCCACTGGCCGACAGGTCCTAAATGACCTC

GGATGGAGAAACTACCAAAATGAAAGTTGTAGATCTCGAAAAGTTATGAAACTTTGCAGT

TGACAACTTTTTCATTTGAAATCGTTTAGGGCGTCAAACAATCAATTTATGTTCAGTTTA

GTATAATATGTGGGGAACCAAAATCCATTATAGGCACATGTGAGCACGAGGTGCAGTGAT

AGAGGAGGCTACGCGCGAGGGGAGGCCGCGGGTTCGAATCCTCGCAGCCGCAAAGCACAT

GATTTTCGAGCCAAAAGTGCCGCGACTTCGTAGTTGACAACTTTTGGATTTGAAATCGTC

TTTTGGGTCATATCGGTTAAATAGGCCGGTCGGTGGCCGACAGGTCCTAAATGACCTCGG

ACGGAGAAACTACCAAAATGAAAGTTGTAGATCCATAAAATTTATGAAACTTCGT

>CL176Contig39

AGGGGGTCCTGCCCGGACAGAGCTCTACGCCTCTCGGGCAAATTACCCTACCTGTGCAGT

TTGGCACGGCAGACCACTTCCGTACCGACTACGTCAACTTTGTGGTCGCTGACTTCGAGG

GCACCTACCATGCTATCCTTGGTCGCCCCGCGATCACCAAATTCATGGCCGTACCTCATT

ACAGGTACTTGGTGCTTAAGATGCCAACGGAAAAGGGAGTCCTCACTCTCAGAGGCAATG

TATTCGTTGCCTATACCTGTGAAGAGGACAGCTTTCGCGTAGCTGAAGCCCACGACCTCT

CCCTGCGCATGGCGGCAACGACGATGGAGGCAAAGAAGACTCCTCCAAACCAGCTAGAAA

TTCCTGAGCTCAAAGCACCATGAAAG

>CL164Contig1

AGTTCGATCTACATGGGATCTCACCTGAATCGCCCCATCTATCCTCCTGAGGAGAAGTTT

GTTTGGTTTCAAACTCCGATTCAAACAGGAGGAGTACGCCATGCTAATGTGCCTTGGATG

ATCCACATCTTCGGGTCAGGCGCTGATGAGCACATTGAACTATCCATGTGGCTGAGAGCC

CTCACAGCCCAGGCACAACGACGCAATTATCAGGGGCGCGCTCTACCACTGAGCTAATAG

CCCGTCGCGCGGGCCTCCCAAAGGGAGGCCTGCTACGCCAAAAGCGAGAAAAACTCCATC

CCTTTCCTTTTGACATCCCCATGCCGCCACACGGGGGGACATGGGGACGTCAAAAAGGGG

ATCCTATCACTATCAACTAATTTGTTCCGACCTAGGATAATAAGCTCATGAGCTTGGTCT

TACTTCACCCTAAACGAAAGAAGACTTCCATATCCAAGTTTAGCTCAGACGTAGCTGCCT

TCTTTTTGGGCGTGAAGCAGTGTCAAACCAAAATACCCAATAAGCATAAGCATTAGCTCT

CCCTGAAAAGGAGGTGATCCAGCCGCACCTTCCAGTACGGCTACCTTGTTACGACTTCAC

TCCAGTCGCAAGCCTAGCCTTAGGCATCCCCCTCCTTACGGTTAAGGGTAATGACTTCAA

ACCTGGCCAGCTCCTATAGTGTGACGGGCGGTGTGTACAAGGCCCGGGAACGGATTCACC

GCCGTATGGCTGACCGGCGATTACTAGCGATTCCTGCTTCATGCAGGCGAGTTGCAGCCT

GCAATCCGAACTGAGGACGGGTTTTTGGAGTTAGCTCACCCTCGCGAGATCGCGACCCTT

TGTCCCGCCCATTGTAGCACGTGTGTCGCCCAGGGCATAAGGGGCATGATGACTTGGCCT

CATCCTCTCCTTCCTCCGGCTTAACACCGGCGGTCTGTTCAGGGTTCCAAACTCATAGTG

GCAACTAAACACGAGGGTTGCGCTCGTTGCGAGACTTAACCCAACACCTTACGGCACGAG

CTGACGACAGCCATGCACCACCTGTGTCCGCGTTCCCGAGGGCACCCCTCTCTTTCAAGA

GGATTCGCGGCATGTCAAGCCCTGGTAAGGTTCTTCGCTTTGCATCGAATTAAACCACAT

GCTCCACCGCTTGTGCGGGCCCCCGTCAATTCCTTTGAGTTTCATTCTTGCGAACGTACT

CCCCAGGCGGGATACTTAACGCGTTAGCTACAGCACTGCACGGGTCGAGTCGCACAGCAC

CTAGTATCCATCGTTTACGGCTAGGACTACTGGGGTCTCTAATCCCATTTGCTCCCCTAG

CTTTCGTCTCTCAGTGTCAGTGTCGGCCCAGCAGAGTGCTTTCGCCGTTGGTGTTCTTTC

CGATCTCAATGCATTTCACCGCTCCACCGGAAATTCCCTCTGCCCCTACCGTACTCCAGC

TTGGTAGTTTCCACCGCCTGTCCAGGGTTGAGCCCTGGGATTTGACGGCGGACTTGAAAA

GCCACCTACAGACGCTTTACGCCCAATCATTCCGGATAACGCTTGCATCCTCTGTCTTAC

CGCGGCTGCTGGCACAGAGTTAGCCGATGCTTATTCCTCAGATACCGTCATTGTTTCTTC

TCCGAGAAAAGAAGTTGACGACCCGTAGGCCTTCCACCTCCACGCGGCATTGCTCCGTCA

GGCTTTCGCCCATTGCGGAAAATTCCCCACTGCTGCCTCCCGTAGGAGTCTGGGCCGTGT

CTCAGTCCCAGTGTGGCTGATCATCCTCTCGGACCAGCTACTGATCATCGCCTTGGTAAG

CTATTGCCTCACCAACTAGCTAATCAGACGCGAGCCCCTCCTTGGGCGGATTTCTCCTTT

TGCTCCTCAGCCTACGGGGTATTAGCAACCGTTTCCAGTTGTTGTTCCCCTCCCAAGGGC

AGGTTCTTACGCGTTACTCACCCGTTCGCCACTGGAAACACCACTTCCCGTTCGACTTGC

ATGTGTTAAGCATGCCGCCAGCGTTCATCCTGAGCCAGGATCGAACTCTCCATGAGATTC

ATAGTTGCATTACTTATAGCTTCCTTATTCGTAGACAAAGCGGATTCGGAATTGTCTTTC

CTTCCAAGGATAACTTGTATCCATGCGCTTCAGATTATTAGCCTGGAGTTCGCCACCAGC

AGTATAGCCAACCCTACCCTATCACGTCAATCCCACAAGCCTCTTATCCATTCCCGTTCG

ATCGTGGTGGGGGAGTAAGTCAAAATAGAAAAAACTCACATTAGGTTTAGGGATAATCAG

GCTCGAACTGATGACTTCCACCACGTCAAGGTGACACTCTACCGCTGAGTTATATCCCTT

CCCCGTCCCATCGAGAAAGAGAATTAACGAATCCTAAGGCAAAGGGGCGAGAAACGCAAG

GCCAGGCCACTCTTCCTCCGGGCTTTCTTTCCGCACTATTATGAACAGTCAAATAATGGG

AAAAATTGGATTCAATTGTCAACCGGTCCTATCGAAAATAGGATTGACTATGGATTCGAG

CCATAGCACATGGTTTCATAAAATCTGTACGATTTTCCCGATCTAAATCGAGTGGGTTTC

CATGAAGAAGATCTTGTTCAGCATGTTCTATTCGATACTGGTAGGAGAAGAACCCGACTC

GGTATTCTTAAAAAAAGAGGGGAAGCAGAACCAAGTCAAGATGATATGGATCGCCCCTTC

TTCTTGCGCCAAAGATCTTACCATTTCTGAAGGAACTGGGGCTACATTTCTTTTCAATTT

CCATTCAAGAGTTTCTATCTGTTTCCACGCCCTTTTTTTGAGACCTCGAAACATGAAATG

GACAAATTCCTTCTCTTAGGAACACATACAAGAAAAAGGATAATGGTAGCCCTCCCATTA

ACT

>CL194Contig1

CTGGTCGTAGGTTCGAATCCTACTTGGGGAGATTTGATTCATTCTTTAATGTAAGAATAA

AGAATTGAATTAAAAGGCTTGCTTTGACCCTTAGGAGTAGGTAACCCGTTCGCTATCCTT

GTTTCTATTGCATTCTATCTCATCGTATCACATTCTGTTCTACGATTCCACTTCGACAAA

AGGAAAGAGCATACCCAAGTTCAATAGCTTTACGTCCGCTATTCCGATCATGATTTTCCT

ACCCTCAGGGAGAAAGTAAAGGTCCTTCCCCCTTTGGAAGGCTGTGGGCGAGGAGGGATT

CGAACCCCCGACACCGTGGTTCGTAGCCACGTGCTCTAATCCTCTGAGCTACAGGCCCAC

CCCGTCTCCACTGGATCTCTTCCCGGGGATACCCCCCAAAAGGAACCTTCTTCTCCTCAG

CCATTTCATTTCGGGTTAAGAAGATGGGAAAGCGCCTTTCTCTCTATAAGAACAGTGCGT

TCTGAGGTGTGAAGTGGGAGAGAGGGGATGATTGAGGTTTTGAATAAGACGACCTTTGTG

TTTTGGATTTGGATCTTTTTCGTATTTCAAAATAGTGAAAAAGTCAAATAAGAGGTGTTA

AGCTTTTTATCATTCTGGCATCGAGCTATTTTGCCGCAGGACCTCCCCTACAGTATCGTC

ACCGCAGTAGAGTTTAACCACCAAATTCGGGATGGATTGGTGTGGTTCCTCTACGCCTAG

GACACCAGAATATCGAACCATGAACGAGAAAAGGCATGAGAGAAATATTGGCTAGTAATT

GTGAAGCCCCAATTCTTAACTGGAAGGGACACCAAAGGACTCTGCCCTCCCTCTCTATTT

ATCCAAGAGATGGAAGGGCAGAGCTTTTTTTTGGTTTTTTCATCTTTTCTTTTCATCAAA

GAGTTGAACAATGAAGATAGATGGCAAGTGCCTGATCGATTTGATCAGGTCGTGTAGGAA

CAAGGTTCAAATCGTTCGTTCGTTAGGATGCCTCAGCTGCATACATCACTGCACTTCCAC

TTGACACCTATTTAAACGGCTCGTCTCGCCGCTACCTTATCCTATTTCCATACTTCTGTC

GCTCCATCCCCGTATGGGTGGAGAACCCGTCGCTGTCTCGGCTGTGCTACCGGAGGCTCT

AGGGAAGTCGGAGGAGAGAGCACTCATCTTGGGGTGGGCTTACTACTTATATGCTTTCAG

CAGTTATCCTCTCCACACTTGGCTACCCAGCGTTTACCGTAGGCACGATAACTGGTACAC

CAGAGGTGCGTCCTTCCCGGTCCTCTCGTACTAGGGAAAGGTCCTCTCAATGCTCTAACG

CCCACACCGGATATGGACCGAACTGTCTCACGACGTTCTGAACCCAGCTCACGTACCGCA

TTAATGGGCGAACAGCCCAACCCTTGGAACCACCTACAGCTCCAGGTGGCGAAGAGCCGA

CATCGAGGTGCCAAACCTTCCCGTCGATGTGGACTCTTGGGGAAGATCAGCCTGTTATCC

CTAGAGTAACTTTTATCCGTTGAGCGACGGCCCTTCCACTCGGCACCGTCGGATCACTAA

GGCCGACTTTCGTCTCTGCTCGACGGGTGAGTCTTGCAGTCAAGCTCCCTTCTGCCTTTG

CACTCGAGGACCAATGTCCGTCTGGCCCGAGGAAACCTTTGCACGCCTCCGTTACCTTTT

GGGAGGCCTACGCCCCATAGAAACTGTCTACCTGAGACTGTCCCTTGGCCCGCGGGTCTG

ACACAAGGTTAGAATCCGAGCTCTTCCAGAGTGGTATCTCACTGATGGCTCGCCCCCCCG

AAGGGGGCCTTCTTCGCCTT
